# Stratified Therapeutic Drug Monitoring Could Potentially Improve the Efficacy and Safety of Oxcarbazepine in Children with Epilepsy: Novel Insights from a Single-Center, Large-Sample, Retrospective Real-World Study

**DOI:** 10.3390/ph19030415

**Published:** 2026-03-03

**Authors:** Yi-Jing Liu, Xi-Li Sun, Yue Li, Xiao-Peng Lu, Chun-Feng Wu, Hu Guo, Feng Chen

**Affiliations:** 1 Pharmaceutical Sciences Research Center, Department of Pharmacy, Children’s Hospital of Nanjing Medical University, Nanjing 210008, China; 2 School of Basic Medicine and Clinical Pharmacy, China Pharmaceutical University, Nanjing 211198, China; 3 Department of Neurology, Children’s Hospital of Nanjing Medical University, Nanjing 210008, China

**Keywords:** oxcarbazepine (OXC), 10,11-dihydro-10-hydroxycarbamazepine (MHD), epilepsy, children, therapeutic drug monitoring (TDM), real-world study, individualized therapy

## Abstract

**Objective**: This study aimed to characterize the population exposure, efficacy, and safety profiles of oxcarbazepine (OXC) in Chinese children with epilepsy using real-world data, define its optimal therapeutic range, and inform individualized therapy. **Methods**: This single-center retrospective cohort study included pediatric patients (<18 years) who received OXC therapy between September 2021 and August 2024, with follow-up continuing until February 2025. The concentration of the active metabolite 10,11-dihydro-10-hydroxycarbamazepine (MHD) in plasma was monitored. A mixed-effects model identified factors influencing MHD exposure. Logistic regression and Cox proportional hazards models were used to analyze the concentration–efficacy relationship, while Kaplan–Meier and time-to-onset analyses were performed to characterize adverse events. **Results**: Among 824 included patients (1976 concentration samples), body weight, age, treatment duration, and epilepsy type significantly influenced MHD’s exposure levels. The 12-month overall response rate was higher in monotherapy than add-on therapy (82.9% vs. 60.4%). A plasma MHD concentration ≥ 10 μg/mL was identified as a critical “risk transition point” for treatment failure (PSM-adjusted OR = 2.42, *p* < 0.001). Multivariate logistic analysis confirmed higher concentrations, specific etiologies, and polytherapy as risk factors for inefficacy. Cox regression further revealed that concentrations ≥ 10 μg/mL and specific etiologies were predictors of reduced long-term treatment persistence. Adverse events occurred in 30.5% of patients; for most, the risk did not change over time. **Conclusions**: This study tentatively proposed a therapeutic reference range (3.0–20.0 µg/mL) of MHD for Chinese children with epilepsy and identified a concentration ≥ 10 μg/mL as a “risk transition point”. The findings provide practical, evidence-based insights for tailoring OXC therapy and managing potential risks.

## 1. Introduction

Epilepsy is one of the most common chronic neurological disorders in childhood. Long-term recurrent seizures not only contribute to cognitive impairment and psychological–behavioral issues but also profoundly impact the quality of life of affected children and impose a substantial burden on their families [1,2]. For pediatric epilepsy, improving long-term outcomes requires early, standardized treatment that addresses the dual goal of effective seizure control and minimal adverse events. In this context, strategies for anti-seizure medication (ASM) selection and optimization remain a central focus of clinical research [3].

Oxcarbazepine (OXC), a 10-keto derivative of carbamazepine, exerts its potent anti-seizure activity primarily through its active metabolite, 10,11-dihydro-10-hydroxycarbamazepine (MHD). Owing to its established efficacy and favorable tolerability profile, OXC is widely recommended globally as the first-line treatment for focal-onset seizures in children [4,5,6]. However, as with other ASMs, OXC exhibits considerable interindividual variability in its pharmacokinetics within the pediatric population. The half-life of plasma MHD is significantly shorter in children than in adults, while its renal clearance is higher, suggesting that children may require higher body weight-based doses to achieve plasma exposure levels comparable to those in adults [7,8]. The significant pharmacokinetic variability of OXC thus limits its utility of fixed dosing, necessitating personalized dosing strategies.

Indeed, therapeutic drug monitoring (TDM) plays a vital role in the personalized treatment of patients with epilepsy [9,10]. Interestingly, research has shown that adverse events associated with OXC are closely correlated with serum MHD concentration, highlighting the importance of TDM for MHD in mitigating adverse events and tailoring OXC dose [11,12]. TDM for OXC is well established and widely adopted in clinical practice. Notably, previous research has predominantly focused on Caucasian populations, proposing a relatively broad reference range for plasma MHD concentrations (3–35 µg/mL) that was largely derived from mixed-age cohorts or adult data [13,14]. Recent efforts have also utilized population pharmacokinetic models to optimize its dosing regimens; however, these models often rely on the aforementioned broad concentration range [15,16]. Impressively, a clear consensus on the target therapeutic concentration range particularly for Chinese children with epilepsy is still lacking.

Furthermore, the association between plasma concentrations of MHD and OXC’s efficacy and safety in children with epilepsy needs to be elucidated, and the relevant influencing factors should be evaluated in real-world clinical settings. For example, when exploring the impact of key clinical factors on MHD exposure levels, most existing studies have failed to account for repeatedly measured data within patients [17,18], potentially overlooking the influence of interindividual variability. Of note, how plasma MHD concentration and other clinical variables collectively determine both immediate treatment response and long-term treatment persistence in pediatric patients are yet to be clearly defined. Meanwhile, although adverse events associated with OXC (e.g., somnolence, dizziness, rash) have been well described, the quantitative relationship between these events and plasma MHD concentration in pediatric patients, as well as the temporal patterns of adverse event occurrence, still remains largely unknown [11,19]. These current gaps represent practical challenges faced by clinicians when tailoring individualized treatment regimens.

Here, this study aimed (1) to characterize the population exposure characteristics of MHD in Chinese children with epilepsy and identify key influencing factors; (2) to assess OXC’s clinical efficacy and analyze how plasma concentrations and other variables affect treatment response and long-term outcomes; and (3) to explore the concentration–adverse event relationships and characterize their temporal distribution. Through integrated exposure–efficacy–safety analyses, this study aimed to establish an evidence base for precise OXC therapy in Chinese children with epilepsy.

## 2. Results

### 2.1. Characteristics of Included Pediatric Patients

**Figure 1:**
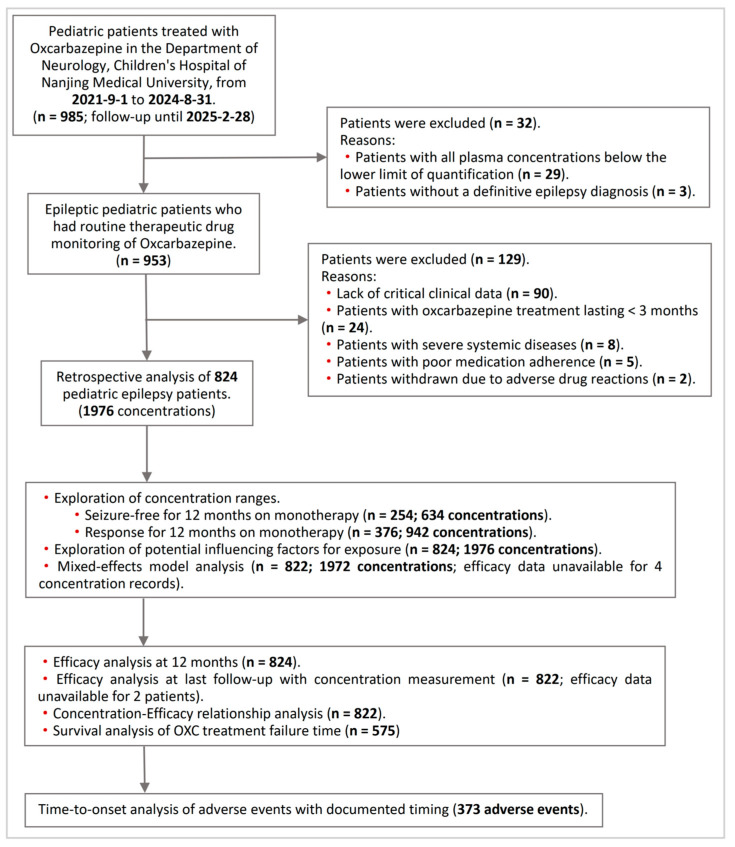
Flowchart of eligible pediatric patients’ selection.

Between 1 September 2021, and 31 August 2024, a total of 985 pediatric patients received either OXC monotherapy or add-on therapy at our hospital. According to the inclusion and exclusion criteria, 161 patients (16.3%) were excluded, resulting in the final cohort of 824 children with epilepsy included in our study (Figure 1). The demographic and clinical characteristics of the patients are presented in Table 1. Among them, 494 were male and 330 were female, with a male-to-female ratio of 1.5:1. The median age at seizure onset (IQR) was 4.77 (5.89) years, while the median age (IQR) at the time of concentration monitoring during follow-up was 8.17 (6.06) years. During follow-up, 101 patients (12.3%) were 2 years of age or younger. The median daily dose of OXC (IQR) was 600 (450) mg/d, and the median daily dose per kilogram of body weight (IQR) was 21.23 (10.29) mg/kg/d. The median plasma MHD concentration (IQR) was 9.75 (6.08) µg/mL.

Prior to starting OXC treatment, EEG reports were available for 446 cases, among whom 416 (93.3%) exhibited abnormal baseline EEG findings. Focal seizures were diagnosed in 464 cases (56.3%), while the seizures in 202 cases could not be definitively characterized and were labeled as unclassified. Half of the children with epilepsy had no other comorbid conditions, and 234 cases (28.4%) presented with developmental delay. Nearly 30% of the children had an identified etiology, primarily structural (14.1%) and genetic (8.1%). Among the included children, 91 patients were found to have genetic abnormalities, of which 67 (73.6%) carried epilepsy-related gene mutation.

### 2.2. Plasma MHD Concentrations

**Figure 2:**
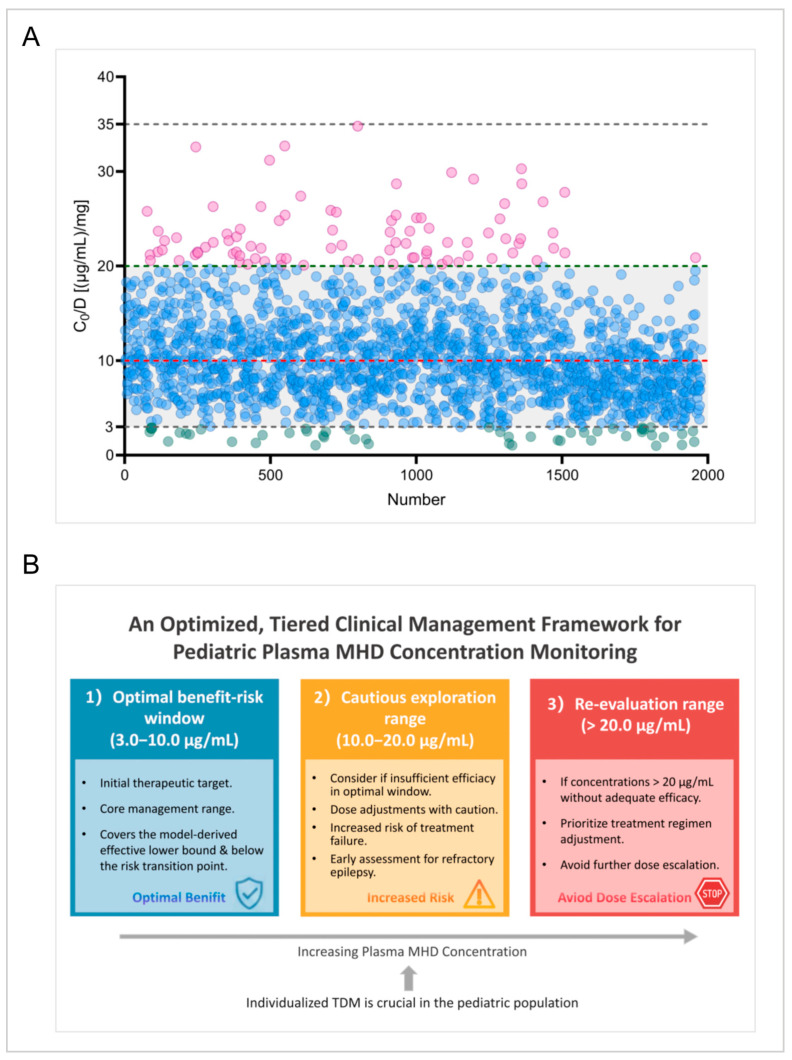
(**A**) Plasma MHD concentrations *C*_0_ (µg/mL) in children with epilepsy, where the *x*-axis shows the number of patients. The upper and lower black dashed lines depict the *C*_0_ reference range recommended by the International League Against Epilepsy (ILAE). The middle green dashed line represents the upper limit of the proposed therapeutic reference range of MHD for pediatric epilepsy patients in this study. The middle red dashed line represents the optimal cutoff value calculated in this study. Circles of different colors represent different ranges of the measured *C*_0_ values in our study: pink circles denote *C*_0_ > 20 µg/mL, blue circles represent *C*_0_ between 3 and 20 µg/mL, and green circles indicate *C*_0_ < 3 µg/mL; (**B**) an optimized, tiered clinical management framework for pediatric plasma MHD concentration monitoring.

In this study, a total of 824 children were included, providing 1976 trough plasma concentration (*C*_0_) data points for MHD monitoring. The concentration distribution ranged from 1.02 to 34.80 µg/mL (Figure 2A). The commonly referenced therapeutic range of 3–35 µg/mL, as proposed by the ILAE [14], is widely adopted in clinical practice. In our center’s routine TDM, 92.9% of the observed *C*_0_ values fell within the range of 3.0–20.0 µg/mL (Figure 2A).

#### 2.2.1. Therapeutic Reference Range in Pediatric Population

We employed a GAMLSS model to estimate the reference range for MHD plasma concentrations in this study. To define the effective concentration range, the modeling data were derived from *C*_0_ values of patients who were on OXC monotherapy and had achieved 12-month seizure freedom or treatment response. Based on the optimal model distribution, the lower (2.5th percentile) and upper (97.5th percentile) limits of the reference interval were calculated. The results showed that the reference ranges (P_2.5_–P_97.5_) for plasma MHD concentrations were 3.16–27.24 µg/mL in the seizure-free group and 3.75–23.33 µg/mL in the treatment response group.

Further stratification by epilepsy type revealed that in children with focal seizures, the reference ranges were 3.82–23.76 µg/mL in the seizure-free subgroup and 3.82–22.94 µg/mL in the treatment response subgroup. These ranges were highly consistent with the TDM-observed range at our center (92.9% of data within 3.0–20.0 µg/mL), which includes all epilepsy types. In contrast, cases with generalized seizures and those with unknown seizure type (whether focal or generalized) exhibited higher lower and/or upper reference limits compared to those with focal seizures, suggesting that these groups might require higher OXC doses or have relatively more refractory conditions. This finding further indicated that OXC might be more suitable for patients with focal seizures. Meanwhile, the reference range for plasma MHD concentrations in patients with unclassified seizures was similar to that of the focal seizure group (Table 2).

#### 2.2.2. Potential Influencing Factors of Plasma MHD Concentration

**Figure 3:**
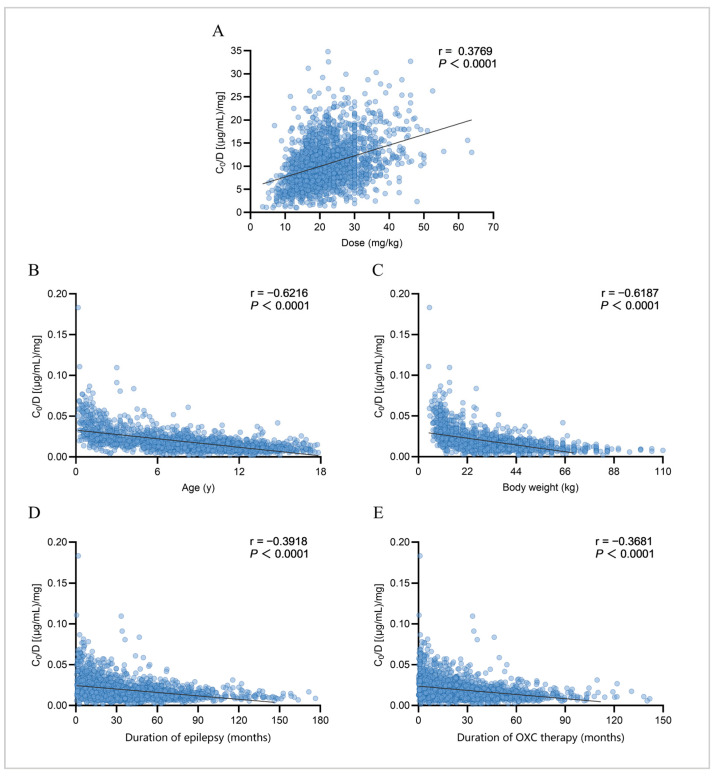
Correlation analysis of MHD *C*_0_ and *C*_0_/Dose ratio [(µg/mL)/mg] with continuous variables. Each blue circle represents an individual patient. The black line represents the trend line. (**A**) Correlation between *C*_0_ and weight-adjusted dose (mg/kg); (**B**) correlation between *C*_0_/Dose ratio and age; (**C**) correlation between *C*_0_/Dose ratio and body weight; (**D**) Correlation between *C*_0_/Dose ratio and duration of epilepsy; (**E**) correlation between *C*_0_/Dose ratio and duration of OXC therapy.

This study analyzed potential influencing factors of plasma MHD concentration based on 1976 blood concentration measurements from the 824 pediatric patients. First, correlation analysis was employed to assess the relationships between continuous variables and exposure factors (Figure 3). A weak to moderate positive correlation was observed between the *C*_0_ value and the corresponding daily OXC dose per kg of body weight (r = 0.3769, *p* < 0.0001; Figure 3A). Among all patients, a significant moderate to strong negative correlation was found between age and the *C*_0_/Dose ratio (r = −0.6216, *p* < 0.0001; Figure 3B). Specifically, the *C*_0_/Dose ratio in children aged 0–≤2 years (230 *C*_0_ measurements; similarly below) who received off-label medication was significantly higher than that in other age groups—1.46, 2.18, and 3.03 times that of children aged 2–≤6 years (396 *C*_0_), 6–≤12 years (998 *C*_0_), and 12–≤18 years (352 *C*_0_), respectively (*p* < 0.0001). Similarly, a significant moderate to strong negative correlation was also identified between body weight and the *C*_0_/Dose ratio (r = −0.6187, *p* < 0.0001; Figure 3C). Additionally, both duration of epilepsy and duration of OXC therapy showed weak to moderate negative correlations with the *C*_0_/Dose ratio (duration of epilepsy: r = −0.3918, *p* < 0.0001, Figure 3D; treatment duration: r = −0.3681, *p* < 0.0001, Figure 3E).

**Figure 4:**
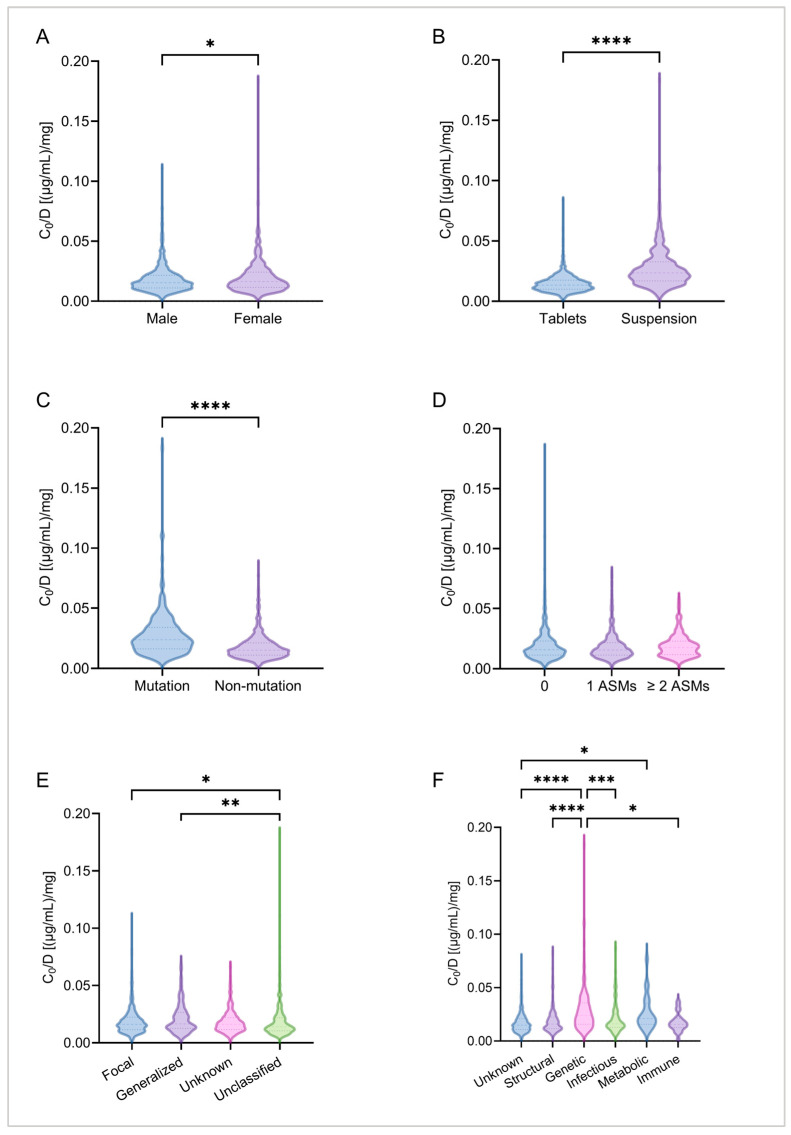
Comparisons of MHD *C*_0_/Dose ratios [(µg/mL)/mg] across different categorical variables. (**A**) Comparison of *C*_0_/Dose ratios by sex; (**B**) comparison of *C*_0_/Dose ratios by dosage form; (**C**) comparison of *C*_0_/Dose ratios by gene mutation; (**D**) comparison of *C*_0_/Dose ratios by number of concomitant medications; (**E**) comparison of *C*_0_/Dose ratios by epilepsy type; (**F**) comparison of *C*_0_/Dose ratios by etiology. *, *p* < 0.05; **, *p* < 0.01; ***, *p* < 0.001; ****, *p* < 0.0001.

Next, differences in *C*_0_/Dose ratios among groups of categorical variables were compared. The results revealed a significant difference in *C*_0_/Dose ratios between sexes, with females exhibiting a 6.17% higher plasma MHD exposure level than males (*p* = 0.0190; Figure 4A). Regarding dosage form, patients taking the OXC oral suspension had a significantly higher *C*_0_/Dose ratio—75.37% higher—than those taking tablets (*p* < 0.0001; Figure 4B). A further subgroup analysis of patients aged ≥ 5 years confirmed that the formulation-based difference remained significant (*p* < 0.0001). ASMs in add-on therapy had no significant effect on the *C*_0_/Dose ratio (*p* = 0.1532; Figure 4D). Significant differences in *C*_0_/Dose ratios were also observed among different epilepsy types, particularly between focal and unclassified seizures (*p* = 0.0128) and between generalized and unclassified seizures (*p* = 0.0079) (Figure 4E). Patients with genetic mutations (whether epilepsy-related or not) had significantly higher *C*_0_/Dose ratios than those without mutations (*p* < 0.0001; Figure 4C). Moreover, cases with genetic etiologies also exhibited significantly higher *C*_0_/Dose ratios compared to other etiology groups (*p* < 0.0001; Figure 4F).

Following this, the mixed-effects model results revealed that age (years), body weight (kg), duration of OXC therapy (months), and epilepsy type had statistically significant effects on plasma MHD concentration (Table 3). Specifically, each one-year increase in age (β = −0.046, *p* < 0.001) or one-kg increase in body weight (β = −0.007, *p* < 0.001) was associated with a significant decrease in the log-transformed *C*_0_/Dose ratio, suggesting that older and heavier children require higher doses to achieve the target blood concentration. Longer OXC treatment duration was also correlated with a reduction in the *C*_0_/Dose ratio (β = −0.002, *p* = 0.014). Furthermore, using focal seizures as the reference, unclassified seizure type was significantly associated with a lower log-transformed *C*_0_/Dose ratio (β = −0.092, *p* = 0.005).

Consequently, the mixed-effects model demonstrated good overall fit. Fixed effects alone explained 41.3% of the variance (marginal R^2^; R^2^m = 0.413), while fixed and random effects together explained 63.0% of the variance (conditional R^2^; R^2^c = 0.630). The intraclass correlation coefficient (ICC) was 0.371, indicating that 37.1% of the total variance was attributable to interindividual differences. Analysis of random effects showed that the variance for individual random intercepts was 0.074 (SD = 0.271), and the residual variance was 0.125 (SD = 0.354), suggesting that unmeasured factors between patients remained a significant source of variation in the *C*_0_/Dose ratio after controlling for fixed effects. Model diagnostics indicated that residuals were approximately normally distributed without heteroscedasticity (Supplementary Figure S1), confirming that the model met basic assumptions and the results were reliable.

To conclude, this study identified multiple patient-specific factors influencing plasma MHD concentration through a mixed-effects model and demonstrated that interindividual variability was the major source of variation in plasma concentration.

### 2.3. Efficacy

#### 2.3.1. Treatment Outcomes at 12 Months

**Figure 5:**
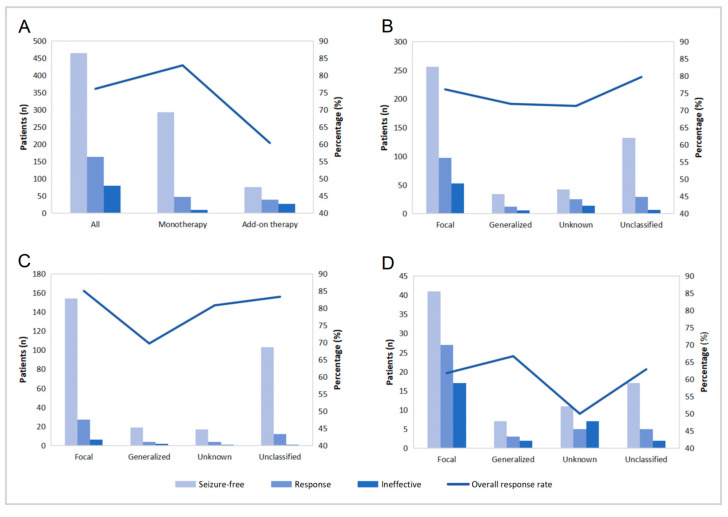
The seizure frequency at 12 months after initiating OXC therapy. (**A**) Divided into all enrolled patients, monotherapy group, and add-on therapy group. (**B**) All enrolled patients stratified by different epilepsy types. (**C**) Monotherapy group stratified by different epilepsy types. (**D**) Add-on therapy group stratified by different epilepsy types.

Prior to starting OXC therapy, 824 pediatric epilepsy patients exhibited varying degrees of seizure activity. Seizure frequency was recorded before and after the start of OXC treatment at 1, 3, 6, 12, 18, 24, 36–48, and >48 months to evaluate therapeutic efficacy. Of note, this study focused on a cross-sectional statistical analysis of the efficacy data at 12 months of treatment (Figure 5).

At 12 months of OXC treatment, the overall response (complete or partial remission) rate was 76.1% (*n* = 627), and 464 patients achieved seizure-free status (Figure 5A). Interestingly, the overall response rate was significantly higher in patients receiving monotherapy (82.9%) compared to those receiving add-on therapy (60.4%) (Figure 5A).

Subsequently, a stratified analysis was performed based on epilepsy type. Among all included patients, the overall response rate at 12 months for those with focal seizures was 76.1% (353/464), which was identical to the rate observed in the total study population (Figure 5B). Within the monotherapy subgroup, OXC treatment yielded a significantly higher overall response rate in patients with focal seizures (85%) compared to those with generalized seizures (69.7%) (Figure 5C). Interestingly, within the add-on therapy subgroup, the overall response rate was slightly higher in patients with generalized seizures (66.7%) than in those with focal seizures (61.8%), although the overall efficacy in this subgroup remained relatively low (Figure 5D).

#### 2.3.2. Analysis of Efficacy at the Last Concentration Monitoring Follow-Up

The clinical efficacy and relevant characteristics of the patients are detailed in Table 4. The treatment response rate among the 822 epilepsy patients was 85.9% (*n* = 706), including 288 females. Among the responders, the median epilepsy duration was 39.9 months, which was similar to that of the non-responder group (median: 40.3 months, *p* = 0.799). However, the median duration of OXC therapy was longer in responders than in non-responders (median: 32.1 months vs. 26.6 months, *p* = 0.042). Additionally, the median dose received by responders was 600.0 mg/d, significantly lower than that of non-responders (787.5 mg/d, *p* < 0.001). Notably, the median plasma MHD concentration in responders was also significantly lower than that in non-responders (9.0 µg/mL vs. 12.0 µg/mL, *p* < 0.001).

Impressively, significant differences in treatment response rates were observed among different etiological subgroups (*p* < 0.001). Specifically, the response rate reached 89.6% in patients with epilepsy of unknown etiology and 86.2% in those with genetic etiology, while relatively lower response rates were observed in patients with metabolic (60.0%) and infectious (67.6%) etiologies. Furthermore, a statistically significant difference was also found between responders and non-responders across different types of epilepsy (*p* = 0.044). At the last concentration monitoring follow-up, 93.6% of cases in the monotherapy group achieved effective seizure control, which was significantly higher than the response rate in patients concurrently using two or more ASMs (62.4%, *p* < 0.001).

Next, after univariate analysis, factors with statistical significance were further included in a multivariate logistic regression analysis. Using the treatment response status at the last concentration monitoring follow-up as the dependent variable, independent variables were selected through a stepwise regression method based on the bidirectional AIC. The final multivariate logistic regression results are shown in Table 5. The epilepsy type was not included in the final model as all its categories lacked statistical significance (*p* > 0.05). Moreover, the AIC value of the model including this variable was 584.46, while the AIC value of the stepwise-optimized model was 579.23, indicating a better model fit and further supporting its exclusion.

Of note, the final model results showed that a higher plasma MHD concentration (OR = 1.10, 95% CI: 1.05–1.15, *p* < 0.001) was significantly associated with an increased risk of treatment inefficacy. Certain specific etiologies were also associated with a higher risk of treatment inefficacy, including structural etiology (OR = 1.99, 95% CI: 1.11–3.50, *p* = 0.019), infectious etiology (OR = 2.75, 95% CI: 1.14–6.30, *p* = 0.019), and metabolic etiology (OR = 4.87, 95% CI: 1.07–20.43, *p* = 0.031). Additionally, concomitant use of one (OR = 2.63, 95% CI: 1.58–4.42, *p* < 0.001) or two or more ASMs in add-on therapy (OR = 6.09, 95% CI: 3.43–9.91, *p* < 0.001) were also significantly associated with an increased risk of treatment inefficacy. In contrast, neither the therapy duration nor the dose of OXC showed statistical significance.

#### 2.3.3. Relationship Between Treatment Response and Plasma MHD Concentration

**Figure 6:**
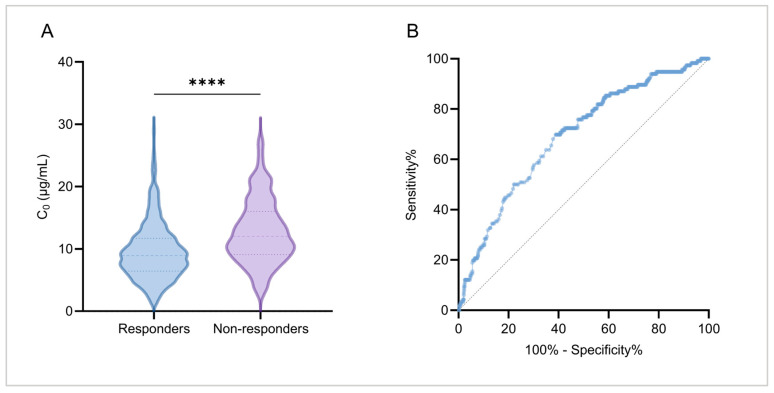
Analysis of efficacy and plasma MHD concentration *C*_0_ (µg/mL). (**A**) Comparison of C0 between responders and non-responders; (**B**) the ROC curve of plasma MHD concentration for predicting treatment response. The ROC curve demonstrates the predictive performance of plasma MHD concentration for treatment response (*n* = 822). The optimal cutoff value was 10.0 µg/mL (sensitivity = 69.8%, specificity = 61.2%). The area under the curve (AUC) was 0.69 (95% CI: 0.64–0.74, *p* < 0.001). The black dashed line indicates random prediction (AUC = 0.5). Interpretation note: The ROC analysis evaluates overall discriminative ability but does not assess model calibration or clinical utility at specific thresholds. ****, *p* < 0.0001.

The aforementioned results indicate a significant association between plasma MHD concentration and OXC treatment efficacy, with the median MHD concentration in responders being significantly lower than that in non-responders (*p* < 0.001; Figure 6A). To further evaluate its clinical relevance, a ROC curve was constructed based on the last plasma concentration monitoring data points and corresponding treatment response status of the 822 cases, aiming to analyze the predictive ability of plasma MHD concentration for OXC treatment response (Figure 6B). The analysis revealed an area under the curve (AUC) of 0.69 (95% CI: 0.64–0.74, *p* < 0.001), indicating a moderate discriminative ability of the model to distinguish between treatment responders and non-responders. The optimal cutoff value, determined using the Youden index method, was 10.0 μg/mL. At this threshold, the model exhibited a sensitivity of 69.8% and a specificity of 61.2%, suggesting that this concentration cutoff value correctly identified 69.8% of real non-responders while accurately distinguishing 61.2% of real responders.

**Figure 7:**
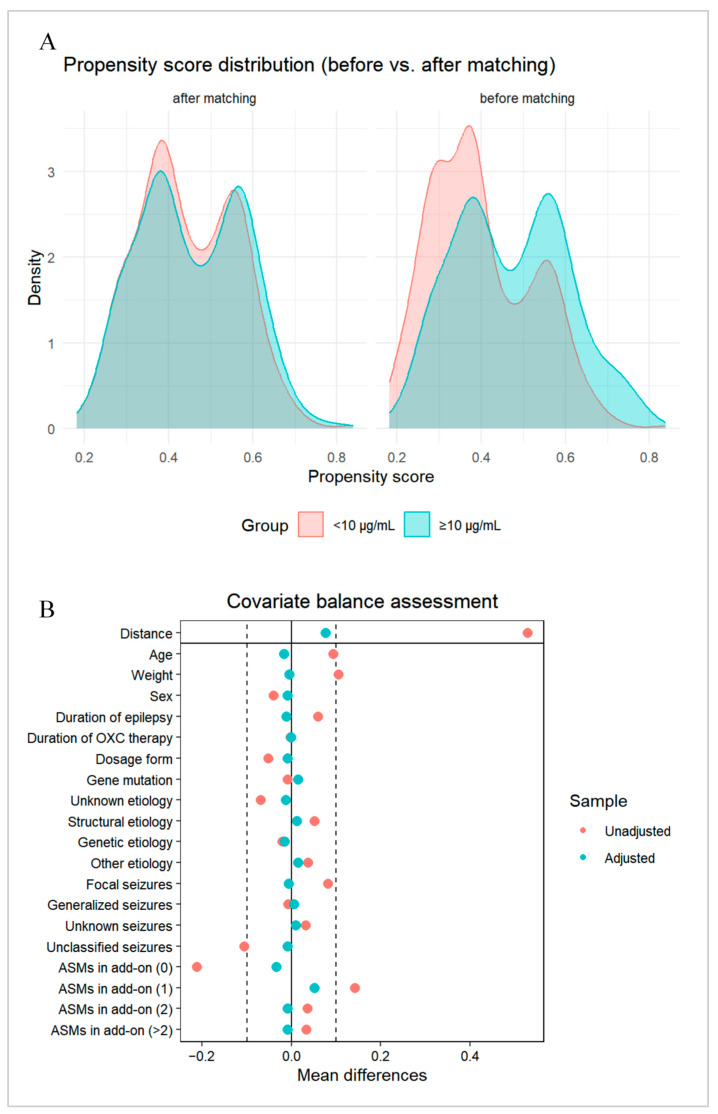
Assessment of propensity score matching quality. (**A**) Propensity score distribution before and after matching; (**B**) covariate balance assessment. The vertical dashed line indicates standardized mean differences (SMD) = 0.10 (balance threshold).

Following this, patients were stratified into two groups based on the optimal plasma MHD concentration cutoff. After controlling for baseline confounders between the groups using PSM, differences in treatment response status were further compared. The demographic and clinical characteristics of the study population before and after matching are detailed in Supplementary Table S1. Evaluation of PSM effectiveness demonstrated that intergroup differences were effectively controlled. Specifically, the distribution of propensity scores became highly consistent between the two groups after matching (Figure 7A), and the standardized mean differences for all covariates were below the threshold of 0.1 (Figure 7B), indicating that the covariates were well-balanced across groups post matching and suitable for subsequent comparative analysis of treatment response differences.

Next, the univariate logistic regression analysis was employed to compare treatment efficacy differences between the two groups according to PSM-balanced baseline characteristics. Statistical analysis revealed significant differences in treatment efficacy between the concentration groups after effectively controlling for baseline confounding factors. Specifically, the treatment response rate was 90.2% (294/326) in the concentration < 10 µg/mL group, compared to 79.1% (258/326) in the concentration ≥ 10 µg/mL group. Patients in the concentration ≥ 10 µg/mL group had a significantly increased risk of treatment inefficacy compared to those in the concentration < 10 µg/mL group (OR = 2.42, 95% CI: 1.55–3.85, *p* < 0.001). This result suggests that after balancing potential confounding factors, the odds of treatment inefficacy in patients with concentrations ≥ 10 µg/mL were 2.42 times higher than those in patients with concentrations < 10 µg/mL.

#### 2.3.4. Survival Analysis of OXC Treatment

We performed survival analysis on follow-up data from 575 pediatric epilepsy patients initially treated with OXC monotherapy to evaluate long-term treatment outcomes. Kaplan–Meier analysis revealed a median time to treatment failure of 73.2 months in this cohort (Supplementary Figure S2). The treatment failure-free survival rates were 85.5% at 12 months, 75.3% at 24 months, 60.3% at 48 months, and 53.7% at 60 months.

First, the Cox proportional hazards regression model was used to identify potential factors influencing time to treatment failure. Univariate analysis indicated that sex, dosage form, and genetic mutation status had no significant effect on time to treatment failure (*p* > 0.05). However, age, body weight, plasma MHD concentration, etiology, and epilepsy type were significantly associated with the risk of treatment failure. As shown in Table 6, patients aged ≥ 9 years (HR = 0.70, 95% CI: 0.53–0.94, *p* = 0.016) and those weighing ≥ 33 kg (HR = 0.68, 95% CI: 0.51–0.91, *p* = 0.009) had a significantly reduced risk of treatment failure, while a plasma MHD concentration ≥ 10 μg/mL (HR = 1.98, 95% CI: 1.49–2.63, *p* < 0.001) was associated with a significantly increased risk. Compared to patients with unknown etiology, those with infectious etiology (HR = 2.56, 95% CI: 1.38–4.75, *p* = 0.003), metabolic etiology (HR = 3.37, 95% CI: 1.07–10.64, *p* = 0.038), other etiology (HR = 2.56, 95% CI: 1.30–5.05, *p* = 0.006), and structural etiology (HR = 1.71, 95% CI: 1.17–2.49, *p* = 0.006) all showed significantly increased risks of treatment failure. Regarding epilepsy types, the hazard ratio was 1.65 (95% CI: 1.09–2.48) for the unknown seizure type group and 0.44 (95% CI: 0.29–0.66) for the unclassified seizure type group. Compared to the focal seizure type group, patients with unknown seizure types had a relatively higher risk of treatment failure (*p* = 0.017), while those with unclassified seizure types were associated with a reduced risk (*p* < 0.001).

Next, factors that showed statistical significance in the univariate analysis were further included in a multivariate Cox proportional hazards regression model (Table 6, Supplementary Figure S3). Multivariate analysis demonstrated that a plasma MHD concentration ≥ 10 μg/mL (HR = 1.73, 95% CI: 1.29–2.32, *p* < 0.001) remained a risk factor for treatment failure, and the risk of treatment failure in this group was 1.73 times that of patients treated with concentrations < 10 μg/mL. Meanwhile, specific etiology types also showed significant predictive value; e.g., compared to the unknown etiology group, patients with infectious etiology (HR = 2.30, 95% CI: 1.23–4.31, *p* = 0.009) and metabolic etiology (HR = 3.74, 95% CI: 1.18–11.85, *p* = 0.025) had relatively increased risks of treatment failure. Additionally, compared to cases with focal seizures, unknown seizure type (HR = 1.58, 95% CI: 1.04–2.40, *p* = 0.031) was also a risk factor for treatment failure, while unclassified seizure type (HR = 0.49, 95% CI: 0.32–0.74, *p* = 0.001) was a protective factor. It was worth noting that age and body weight, which were significantly associated in the univariate analysis, lost statistical significance after multivariate adjustment (*p* > 0.05). Furthermore, although other etiology (*p* = 0.076) and structural etiology (*p* = 0.060) showed an increasing trend in risk, they did not reach statistical significance.

### 2.4. Adverse Events

Among the cases included in this study, a total of 251 (30.5%) reported at least one adverse event (Table 7). The most commonly reported adverse events included dizziness/headache (*n* = 88), gastrointestinal disturbance (*n* = 74), and rash (*n* = 49). Additionally, the incidence rates of somnolence and psychiatric symptoms were relatively high, at 5.2% and 5.1%, respectively. Notably, three cases exhibited suicidal ideation or self-injurious behavior; one of them attempted suicide during dose reduction, while the other two displayed self-injury tendencies or intense harmful thoughts during treatment.

#### 2.4.1. Relationship Between Adverse Events and Plasma MHD Concentration

Based on TDM and adverse event follow-up data from 824 pediatric epilepsy patients, we firstly conducted an exploratory analysis of the correlation between adverse events and plasma MHD concentrations. A statistically significant difference was observed in plasma MHD concentrations between patients with and without adverse events (*p* < 0.001; Supplementary Figure S4A). Further analysis was performed on data from patients who received OXC monotherapy throughout the course. The median MHD *C*_0_ in patients who experienced adverse events remained significantly higher than that in those without adverse events (9.3 µg/mL vs. 8.3 µg/mL, *p* = 0.001; Supplementary Figure S4B).

Subsequently, a ROC curve was plotted to further evaluate the predictive performance of plasma MHD concentration for adverse events. The results indicated that its predictive value was highly limited. Among all included patients (*n* = 824), the AUC was only 0.55 (95% CI: 0.52–0.57, *p* < 0.001; Supplementary Figure S4C). In the OXC monotherapy subgroup (*n* = 387), the AUC was 0.57 (95% CI: 0.53–0.61, *p* = 0.001; Supplementary Figure S4D). Both AUC values were close to 0.5, the reference line for random guessing, indicating that plasma MHD concentration had poor discriminative ability for predicting the occurrence of adverse events. The optimal cutoff value calculated based on the Youden index was 8.68 µg/mL in both groups; however, its predictive performance was suboptimal (all-patient group: sensitivity 66.4%, specificity 42.9%; monotherapy group: sensitivity 60.8%, specificity 54.1%). This cutoff value might lead to a high proportion of false-positive and false-negative results. Thus, while a statistically significant association may exist, it does not translate to clinically meaningful predictive utility. In conclusion, based on the current data, plasma MHD concentration did not appear to be a reliable indicator for predicting OXC-related adverse events.

#### 2.4.2. TTO Analysis

**Figure 8:**
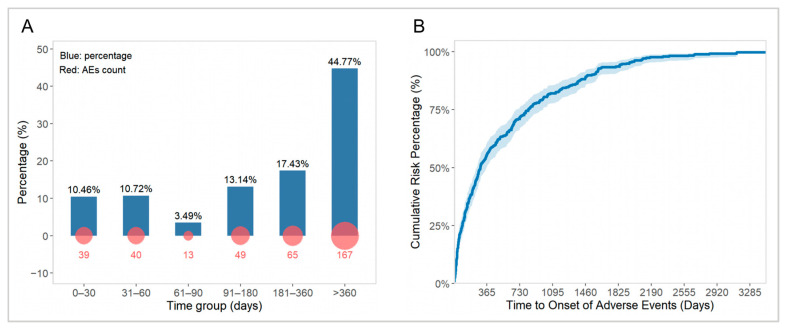
Time-to-onset analysis of adverse events at the overall level. (**A**) Time-to-onset grouping of adverse events. The blue section above represents the percentage of adverse events, while the size of the red circles below indicates the number of adverse events; (**B**) cumulative risk curve of adverse events (median time-to-onset: 295 days).

A TTO analysis was performed on 373 adverse events with clearly documented onset times. Figure 8 illustrates the overall TTO distribution of adverse events in children treated with OXC during the treatment period. The results indicated that the median time to adverse event occurrence was 295 days (95% CI: 253–364; Figure 8B). Within the first month after starting OXC treatment, 39 adverse events were reported, accounting for 10.46% of the total adverse events. Thereafter, the proportion of adverse events reached its lowest level at the third month (3.49%), while long-term adverse events occurring after more than 360 days of treatment accounted for the highest proportion, at 44.77% (Figure 8A).

**Figure 9:**
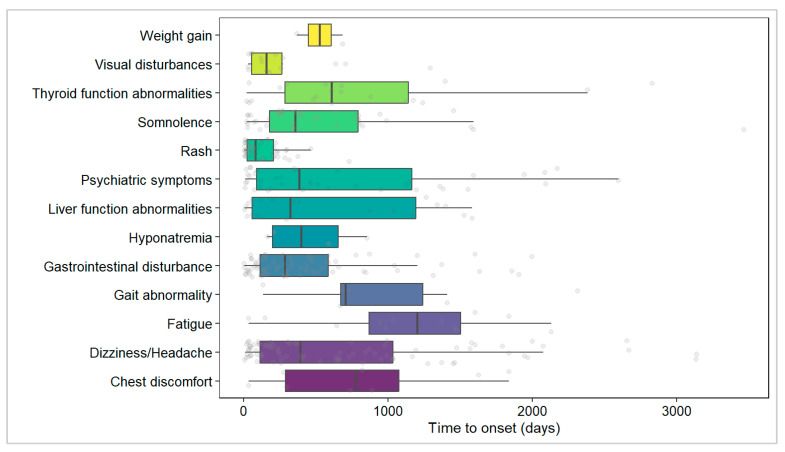
Time-to-onset (TTO) distribution of different types of adverse events. Each circle represents a case of adverse event in this study.

Subsequently, we analyzed the temporal distribution characteristics of different types of adverse events (Figure 9). The results showed that rash occurred relatively early and exhibited a concentrated distribution (median time: 81 days, 95% CI: 49–165), followed by visual disorders (median time: 159 days, 95% CI: 59–637), though the wide CI suggests considerable interindividual variability. In contrast, gastrointestinal disorders (median time: 286 days, 95% CI: 174–438), somnolence (median time: 357 days, 95% CI: 263–637), and dizziness/headache (median time: 392 days, 95% CI: 245–744) predominantly occurred during the mid-to-late stages of treatment. Notably, liver function abnormalities (median time: 323 days, 95% CI: 99–1264) and psychiatric symptoms (median time: 385 days, 95% CI: 184–952) also exhibited wide CIs, reflecting high variability in the timing of these adverse events. Fatigue had the latest median TTO (1203 days), but the upper limit of its CI could not be estimated, indicating significant temporal heterogeneity. Additionally, due to insufficient sample size (*n* < 10), the median TTO for adverse events such as chest discomfort, gait abnormality, hyponatremia, and weight gain should be interpreted with caution.

Next, further analysis of the TTO distribution for various adverse events was conducted using the Weibull shape parameter test (Table 8). The results revealed that only body weight gain (β = 3.84, 95% CI: 1.21–12.20) had a β > 1 and a lower 95% CI limit > 1, indicating a wear-out failure model characterized by a significantly increasing risk over time. This finding suggested that long-term medication use might lead to cumulative risk of this adverse event. For the remaining adverse events, the point estimates of β were close to 1, and their CIs included 1, supporting a random failure model, meaning the risk of these events remained relatively constant over time. It was worth noting that within the random failure model, different adverse events still exhibited distinct temporal tendencies. For instance, rash (α = 126.12, β = 0.90) and visual disorders (α = 265.65, β = 0.91) tended to occur early, while fatigue (α = 1238.24, β = 1.58) and gait abnormality (α = 1090.02, β = 1.43) were more likely to occur later. It should be noted that parameter estimates for some adverse events with small sample sizes (e.g., hyponatremia, body weight gain; *n* < 10) might carry considerable uncertainty and should be interpreted cautiously.

## 3. Discussion

To the best of our knowledge, this represents the first retrospective real-world clinical study conducted in a pediatric epilepsy cohort in China involving 824 participants. Its findings have, for the first time, defined the target concentration range for TDM of MHD, OXC’s active metabolite, and provided insights into patient stratification for MHD monitoring. The study identified key determinants significantly influencing the systemic exposure and efficacy of OXC while also delineating its safety profile. This clinical investigation focusing on Chinese children with epilepsy has yielded some innovative findings, which will be elaborated and discussed in detail in the subsequent sections.

### 3.1. Defining the Stratified Target Concentration Range of MHD Tailored to Chinese Children with Epilepsy

A major finding of this real-world clinical study was the proposal of a plasma concentration reference range for MHD (3.0–20.0 µg/mL) that was better aligned with the specific characteristics of Chinese children with epilepsy. The widely applied therapeutic range (3–35 µg/mL) for MHD in clinical practice originates from the 2008 guidelines for TDM issued by the ILAE Commission on Therapeutic Strategies [14]. This target range has since been adopted in the majority of subsequent studies [9,20,21,22]. It should be noted that such a reference range was primarily derived from adult data [14]. Given the significant physiological and metabolic differences between children and adults [23], the applicability of this range directly to pediatric patients, particularly Chinese children with epilepsy, remains uncertain.

This study firstly integrated descriptive statistics, GAMLSS modeling, and survival analysis to comprehensively evaluate the appropriate therapeutic concentration range of MHD in Chinese pediatric epilepsy patients. The results revealed that 92.9% of the observed *C*_0_ of MHD in children receiving maintenance OXC therapy naturally fell within the 3.0–20.0 µg/mL interval (Figure 2A), which was significantly lower than the upper limit recommended by the ILAE guidelines. Further estimation using the GAMLSS model revealed that the plasma concentration ranges for the overall treatment response group and the focal seizure subgroup were 3.75–23.33 µg/mL and 3.82–22.94 µg/mL, respectively, with the 97.5th percentiles not exceeding 24 µg/mL, showing high consistency with the measured ranges. These results highlighted that the current guideline’s upper limit of 35 µg/mL may far exceed the maximum effective concentration required for most pediatric cases.

Next, this study found that MHD concentration has a certain predictive value for treatment response (AUC = 0.69, *p* < 0.001). This finding was consistent with previous research concluding that serum MHD levels were significantly associated with improved seizure outcomes [24]. Interestingly, multivariate analysis identified a significant “risk transition point”: compared to children with concentrations < 10 µg/mL, those with concentrations ≥ 10 µg/mL had a significantly increased risk of treatment inefficacy (OR = 2.42, *p* < 0.001) and were 1.73 times more likely to experience treatment failure (HR = 1.73, *p* < 0.001). In other words, increasing the dosage to elevate systemic exposure to MHD does not yield additional therapeutic benefits but instead raises the risk of treatment non-response or even failure. This phenomenon underscores the complexity of pharmacological management in pediatric epilepsy.

Indeed, we initially hypothesized that this phenomenon might be related to adverse events induced by higher plasma concentrations; however, subsequent analyses did not support this. Children experiencing adverse events had a significantly higher median plasma MHD concentration (9.3 µg/mL) than those without (8.3 µg/mL), However, ROC curve analysis confirmed that plasma concentration showed limited predictive power for adverse events (AUC ≈ 0.55, see Supplementary Figure S4) and was not a reliable predictor either. Of note, cases with more frequent seizures or refractory epilepsy often receive higher OXC doses, leading to overrepresentation of inherently poor responders in the high-concentration group. For these children, treatment failure may primarily reflect the refractory nature of their epilepsy rather than the elevated plasma concentration itself. Moreover, statistical models may inadequately account for complex disease severity factors, possibly resulting in a spurious association between high concentrations and increased risk. Thus, the observed association in this study was more likely attributable to “confounding by disease severity”.

Based on the above novel findings, we tentatively proposed an optimized, tiered clinical management framework for plasma MHD concentration monitoring (Figure 2B), underscoring the importance of individualized TDM in the pediatric population: (1) Optimal benefit–risk window (3.0–10.0 µg/mL): This interval, covering the model-derived effective lower bound and remaining below the risk transition point, should serve as the initial therapeutic target and core management range for all children; (2) Cautious exploration range (10–20 µg/mL): If efficacy was insufficient within the optimal window, dose adjustments into this range may be considered cautiously, with awareness of the associated increased risk of treatment failure and early assessment for features of refractory epilepsy; (3) Re-evaluation range (>20 µg/mL): When concentrations exceeded 20 µg/mL without adequate efficacy, adjusting the treatment regimen should be prioritized over further dose escalation.

### 3.2. Unveiling the Marked Interindividual Variability in MHD Plasma Concentrations and Its Key Determinants

One major strength of the present study was its inclusion of 824 participants, a substantial cohort that facilitates stratified analyses across multiple research objectives. For example, we had the capacity to monitor plasma MHD concentrations, thereby enabling the evaluation of the impact of various variables on MHD exposure levels in children. Impressively, the study employed a mixed-effects model to handle repeatedly measured drug concentration data, allowing for a systematic analysis of potential factors influencing MHD concentrations and their sources of variation.

First, children are in a continuous state of physiological and functional development, which results in pharmacokinetic characteristics that differ significantly from those of adults [23,25]. This is a critical factor that must be considered for achieving precision therapy. Our analysis revealed that several fixed effects had a significant impact on the *C*_0_/Dose of MHD in children, which is consistent with previous studies reporting variability in the pharmacokinetics of OXC and its active metabolite MHD [17,18,26].

Of note, this study found that as age and body weight increased, the *C*_0_/Dose of MHD decreased significantly (Table 3). This phenomenon may be related to dynamic changes during the ontogeny of various enzymes (such as carbonyl reductase and UGT enzymes) and drug transporters (such as ABCB1 and ABCC2) involved in OXC’s disposition [27,28].

Second, the induction of CYP3A4 by OXC may also contribute to its own clearance to some extent [29,30]. It is noteworthy that, in this study, the duration of OXC therapy and the type of epilepsy were also found to have significant effects on *C*_0_/Dose. Long-term medication may induce metabolic enzyme activity, thereby accelerating the drug’s own clearance and leading to a decrease in plasma concentration [30].

Third, underlying neurophysiological or biochemical pathway abnormalities associated with different types of epilepsy might affect the processes of drug absorption, distribution, metabolism, or excretion, thereby contributing to pharmacokinetic variability. However, the effect sizes (β values) for these two factors were relatively small, particularly for the duration of OXC therapy, suggesting that their clinical significance may be limited or that they represent the cumulative effect of multiple minor influences, warranting further investigation.

The statistical analysis approach also warrants discussion here, given its pivotal role in identifying and confirming sources of variation in plasma MHD concentrations. Intriguingly, a mixed-effects model allows for the simultaneous quantification of the contributions of both fixed and random effects, thereby providing a more comprehensive understanding of the factors affecting drug concentrations. The analysis revealed that the variation in plasma MHD concentrations was primarily attributable to significant interindividual variability. Notably, fixed effects—including measurable clinical factors such as age, body weight, treatment duration, and epilepsy type—individually explained 41.3% of the variation (R^2^m = 0.413). However, the proportion of explained variation increased to 63.0% (R^2^c = 0.630) when both the fixed and random effects (i.e., individual patients) were included in the model. Furthermore, the calculated ICC value was 0.371, indicating that 37.1% of the total variance could be attributed to inherent differences between individual patients. This finding suggested that, in addition to known clinical factors, the random interindividual differences constituted a substantial component of the variability in plasma MHD concentrations. Indeed, considerable variability in drug disposition capacity remained among pediatric patients even after strictly controlling for covariates such as age and body weight.

Interestingly, the random-effects analysis further supported the above findings. The variance for the random intercept of individual patients was 0.074 (SD = 0.271), and the residual variance was 0.125 (SD = 0.354). This indicated that, after excluding the influence of fixed effects, unmeasured factors between patients remained a significant source of variation in the concentration-to-dose ratio. These unmeasured factors might include genetic polymorphisms, unmodeled physiological or pathological conditions, dietary habits, and medication adherence, among others. For example, previous studies have reported that *ABCC2* genetic polymorphisms could affect the pharmacokinetics of OXC in children, leading to interindividual variability in drug exposure [31]. Thus, the findings of this study highlighted the necessity of implementing TDM and individualized dosing for pediatric patients with epilepsy.

### 3.3. Delineating the Clinical Efficacy of OXC and Identifying Its Critical Influencing Factors

Impressively, this study confirms that OXC demonstrated favorable clinical effectiveness in a real-world pediatric epilepsy cohort. The 12-month overall response rate was 76.1%, which is largely consistent with previous reports, thereby further consolidating its role in pediatric epilepsy management [32,33]. Moreover, the overall response rate was significantly higher in patients on monotherapy (82.9%) compared to those on add-on therapy (60.4%), which might partly reflect that the add-on therapy itself represented a more treatment-resistant epilepsy subgroup (Figure 5).

Interestingly, we observed that treatment modality and epilepsy type jointly served as key factors for efficacy stratification. Among children receiving monotherapy, on the one hand, the overall response rate was significantly higher for focal seizures (85%) than for generalized seizures (69.7%), aligning with conclusions from prior studies [24,34,35]. This provided evidence from a Chinese pediatric population supporting OXC’s clinical positioning as a preferred agent for focal epilepsy. Within the add-on therapy subgroup, on the other hand, the overall response rate was slightly higher for generalized seizures (66.7%) compared to focal seizures (61.8%). However, given the overall lower efficacy in this subgroup, such difference should not be directly interpreted as superior efficacy of OXC against generalized seizures, as it may be influenced by confounding factors such as drug interactions or etiology. Previous studies have also observed that OXC therapy retains some efficacy as an add-on therapy in drug-resistant focal epilepsy [36,37], suggesting its potential value in combination therapy.

Next, a multivariate logistic regression model analysis was constructed in order to thoroughly assess individual factors influencing treatment response (Table 5). The results showed that a higher plasma MHD concentration was significantly associated with an increased risk of treatment failure (OR = 1.10, 95% CI: 1.05–1.15, *p* < 0.001). As discussed earlier, this finding suggested that MHD concentration was not “the higher, the better,” but rather implied the existence of a “therapeutic window”. The model analysis further identified etiology as a critical factor affecting efficacy. Structural, infectious, and metabolic etiologies were all significantly associated with an increased risk of treatment failure (*p* < 0.05), potentially related to the severe brain dysfunction, with multiple drug-resistance mechanisms often accompanying these etiologies [38,39]. An additional factor warranted further elaboration. The number of concomitant ASMs was significantly positively correlated with the risk of treatment failure (1 concomitant ASM: OR = 2.63; ≥2 concomitant ASMs: OR = 6.09), further demonstrating that polytherapy may lead to complex drug interactions and unwanted clinical outcomes. For instance, as a CYP3A inducer, OXC may affect the metabolic clearance of co-administered drugs [29,40]. Furthermore, the need for polytherapy itself is a hallmark of drug-resistant epilepsy, and the likelihood of achieving seizure freedom with subsequent pharmacological treatments decreases significantly as the number of previously failed treatment regimens increases [41]. Therefore, polytherapy should be applied with careful consideration of drug interactions and the patient’s drug-resistant background.

Finally, our study supported the implementation of TDM in stratified patients. In the initial monotherapy population, multivariate Cox regression analysis indicated that a plasma MHD concentration ≥ 10 μg/mL was a risk factor for treatment failure (HR = 1.73, 95% CI: 1.29–2.32, *p* < 0.001). Consistent with both multivariate logistic regression and PSM-adjusted analyses, the findings of the Cox model analysis suggested a significantly increased risk of treatment failure in children requiring higher drug concentrations to maintain treatment. The Cox model also confirmed specific etiologies like infectious and metabolic types as predictors of reduced treatment persistence. Thus, early identification and the adoption of more aggressive or targeted treatment strategies are crucial for children with these specific etiologies [42].

### 3.4. Revealing the Clinical Safety Profile of OXC

The safety of OXC in children with epilepsy should be accorded equal importance as its clinical efficacy. This study was not limited to reporting the incidence of adverse events but further explored their association with drug concentrations. Notably, utilizing TTO analysis and Weibull Shape Parameter model fitting, the distribution patterns and temporal characteristics of adverse event onset were revealed. Approximately 30.5% of the pediatric patients reported at least one adverse event, with dizziness/headache, somnolence, gastrointestinal disturbance, or rash being the most common, consistent with previous literature reports [32,37,43]. Some studies have observed that OXC therapy is associated with a higher incidence of hyponatremia and other adverse reactions [35,44,45]. However, in our study, only 4 cases (0.5%) developed hyponatremia (Table 7).

Evaluating the correlation between OXC’s safety and exposure levels of MHD represents a notable aspect of this study. Interestingly, this study found that although the median plasma MHD concentration in patients who experienced adverse events was significantly higher than in those who did not, the area under the ROC curve was only 0.55, indicating that the exposure level has limited discriminative ability for predicting the overall risk of adverse events (Supplementary Figure S4). This finding differed from some previous studies. For example, Striano et al. reported that when the serum MHD concentration ≥ 30 μg/mL, the risk of adverse events significantly increased [19]. A potential reason for this discrepancy may lie in the plasma concentration distribution characteristics of the study population: only 5 cases in our study had MHD concentrations > 30 μg/mL, with the majority having low to moderate exposure levels. This suggested that physicians in our hospital may have proactively adjusted doses to maintain plasma concentrations within a relatively safe range for most cases.

Next, for the first time in a large pediatric population, this study systematically assessed the temporal distribution patterns of OXC adverse events through TTO analysis. Notably, over 44% of adverse events occurred after one year of treatment, with a median TTO of 295 days (Figure 8). This finding suggested that long-term tolerance management for OXC should be considered as clinically important as the initial phase. Further Weibull model fitting of the time distribution for various types of adverse events revealed that, except for body weight gain which conformed to the “wear-out failure” model (i.e., risk increases over time), the majority of adverse events followed the “random failure” model, meaning that their occurrence risk remained relatively constant over time (Table 8). It should be noted that body weight gain is a known adverse reaction of long-term OXC treatment [6]. The results of this study are consistent with this understanding. Indeed, the TTO analysis provided an important practical basis for continuous safety monitoring throughout the entire treatment cycle.

Impressively, within the overall “random failure” framework, different types of adverse events still exhibited distinct temporal tendencies (Figure 9). For example, rash and visual disorders tended to occur early in treatment, consistent with immune-mediated rapid responses or early adaptation mechanisms of the nervous system, and aligning with conclusions from several studies that observing rash predominantly in the initial treatment phase [46,47]. Conversely, adverse events such as psychiatric symptoms in the pediatric population occurred later and exhibited considerable interindividual variability. Indeed, Li et al. reported that although the TTO for OXC-induced psychiatric adverse events was less than 100 days, its TTO distribution was still relatively late compared to other ASMs [48].

Among these psychiatric adverse events, suicidal ideation and self-injurious behavior warrant particular attention due to their severe clinical implications. In our cohort, three patients (0.4%) exhibited such behaviors, with timing ranging from during active treatment to the dose reduction phase. This finding underscores the need for sustained vigilance and proactive monitoring of emotional/behavioral changes throughout OXC therapy, alongside clear risk communication and established intervention protocols to ensure safety.

One more question warranted further discussion. Although a shape parameter (β) close to 1 required careful interpretation, it more likely reflected the complexity of real-world clinical practice rather than negating the association. During long-term treatment, time-varying confounders—such as comorbidities, concomitant medications, or changes in patient physiological status—might mask the potential time-dependent signal of the drug itself, causing the overall distribution pattern to approximate random failure. Therefore, the “random failure” model precisely highlighted the challenges in identifying the temporal distribution patterns of drug adverse events in a real-world setting.

### 3.5. Study Limitations

However, this study had several limitations. First, as a single-center retrospective study, it inherently carried potential limitations such as selection and indication bias (particularly in TDM sampling), unmeasured confounding factors, and heterogeneity in clinical records. Moreover, the generalizability of the conclusions needs to be further validated in multicenter prospective studies. Second, although 824 children with epilepsy were included, the number of patients at each time point varied dynamically due to differences in treatment cycles and the fact that the data were derived from real-world clinical records rather than prospective seizure diaries. Therefore, we focused on analyzing the efficacy data of OXC monotherapy or add-on therapy at the 12-month mark and further conducted a stratified analysis based on epilepsy types. Third, the reliance on electronic medical records rather than standardized assessment instruments introduced specific limitations: the potential for underreporting of adverse events or delays in their documentation, and the absence of standardized metrics for seizure severity or quality of life, which constrained the granularity of outcome measures. Fourth, although genetic mutation status was reported for a subset of patients, the lack of systematic pharmacogenomic integration precluded an analysis of how specific genetic variants may modify drug exposure-response relationships. Fifth, as the findings were derived from an Asian population, their generalizability to non-Asian populations may be limited and requires further validation in multicenter, prospective studies.

Nevertheless, this study, based on a large-sample cohort design, effectively enhanced statistical power through an adequate sample size. The real-world treatment patterns observed in this study, compared to randomized controlled trials, more accurately reflected the use of OXC in routine clinical practice. Furthermore, this study provided a systematic evaluation of exposure to MHD, clinical efficacy, and safety of OXC in pediatric epilepsy patients. Through an in-depth analysis of exposure–efficacy relationships and safety profiles, it offers robust evidence to support the development of individualized treatment strategies and the implementation of therapeutic drug monitoring.

### 3.6. Clinical Significance and Future Perspectives

The proposed stratified management framework in this study holds significant implications for the design of future prospective research and the updating of clinical practice guidelines. First, this study provides a clear hypothesis for subsequent validation through prospective investigations. Comparing the “TDM-based tiered framework” with the traditional “body weight-based empirical dosing approach” could validate the utility of this strategy in terms of efficacy, safety, and long-term clinical outcomes. Second, our analysis highlights mechanistic questions that warrant further exploration in the future. In particular, elucidating the mechanisms underlying the cautiously explorable range (10–20 μg/mL) proposed in this study is crucial—a range within which efficacy plateaus while the risk of treatment failure increases. This observation suggests that future studies should focus on exploring underlying pharmacogenomic, metabolomic, and other mechanisms to explain why some pediatric patients experience reduced efficacy when MHD concentrations reach ≥10 μg/mL. Such insights would provide molecular-level evidence to address clinical decisions regarding “when to switch treatment strategies rather than blindly increasing doses,” thereby identifying patient subgroups who may genuinely benefit from higher drug exposure. Finally, our large-sample real-world evidence offers evidence-based references for updating and refining relevant clinical guidelines. By systematically quantifying exposure–efficacy–safety relationships and demonstrating that substantial interindividual variability is a dominant factor influencing exposure outcomes, this study strongly supports the incorporation of not only routine TDM but also evidence-based individualized target concentration ranges into future guideline recommendations. These attempts and efforts will facilitate a shift from empirical dosing to pharmacokinetics-guided precision medicine in clinical practice.

## 4. Methods

### 4.1. Study Participants

This retrospective study consisted of patients under 18 years of age who were diagnosed and treated with OXC in the Department of Neurology at the Children’s Hospital of Nanjing Medical University from 1 September 2021 to 31 August 2024. All participants were followed up until 28 February 2025. The classification of epileptic seizures was based on the 2025 edition of the guidelines updated by the International League Against Epilepsy (ILAE) [49] and was diagnosed by experienced neurologists.

Exclusion criteria were as follows: (1) received OXC treatment but lacked routine TDM data, without available concentration data or concentrations below the lower limit of quantification; (2) missing or incomplete key medical record information in the hospital information system (HIS); (3) poor medication adherence or self-discontinuation of treatment; (4) discontinuation due to adverse drug reactions; (5) cases lost to follow-up or for other reasons, with less than 3 months of efficacy evaluation; (6) the presence of underlying systemic diseases, severe progressive central nervous system disorders, serious circulatory diseases, or hematologic diseases.

We systematically collected patients’ demographic information, including basic characteristics such as sex, age at seizure onset, and body weight. Additionally, we meticulously organized and documented clinical features, including medical history, pharmacotherapy details, and laboratory monitoring results. These comprised epileptic seizure type, seizure frequency, duration of epilepsy, etiology, comorbidities, electroencephalography (EEG) reports, magnetic resonance imaging (MRI) reports, drug dosage, formulation, blood sampling time, plasma MHD exposure levels, duration of OXC therapy, number and types of both previous and currently concomitant ASMs, and adverse events.

The study protocol was approved by the Ethics Committee of the Children’s Hospital of Nanjing Medical University (Protocol number 202408041-1). As this was a retrospective study design, written informed consents were waived. This study was conducted in accordance with the ethical principles of the Declaration of Helsinki.

### 4.2. Treatment of OXC and Routine TDM for Plasma MHD

All eligible children with epilepsy received either OXC monotherapy or adjunctive therapy, administered orally (as tablets or oral suspension). The initial dose was 8–10 mg/kg/d, divided into two administrations. Depending on clinical response, the daily dose could be increased at intervals of no less than 7 days, with each increment not exceeding 10 mg/kg/d. The final maintenance dose was determined based on clinical efficacy, tolerability, and individual body weight, though the maximum dose generally did not exceed 60 mg/kg/d.

Upon reaching the expected steady state under the maintenance OXC regimen, venous blood samples (2–3 mL) were collected from each patient in anticoagulant tubes the next morning prior to the scheduled dose. The whole-blood samples were routinely transported to our laboratory for TDM of OXC in the included cases. After gentle inversion to ensure thorough mixing, the samples were centrifuged at 4000 rpm for 5 min to obtain plasma supernatant. The plasma concentration of MHD was determined using a previously established and validated LC-MS/MS method by our group [50], which allows for simultaneous quantification of 15 antiepileptic drugs in human plasma. The LC-MS/MS analysis was conducted using a Triple Quad™ 4500MD mass spectrometer (AB Sciex Pte. Ltd., Singapore) coupled with a Jasper™ liquid chromatography system (AB Sciex Pte. Ltd., Singapore). The chromatographic system was equipped with a binary pump (Sciex Dx™), an online degasser (Sciex Dx™), an autosampler (Sciex Dx™), and a column oven (Sciex Dx™). Ionization was achieved using a Turbo V™ ion source via electrospray ionization (ESI).

We utilized the measured concentration data points to further explore the range of plasma MHD concentrations in children and their potential influencing factors. The reference concentration range of MHD was investigated separately in two distinct patient populations: (1) patients who achieved seizure freedom during 12 months of OXC monotherapy, and (2) patients who experienced a 50% or greater reduction in seizure frequency or severity during 12 months of OXC monotherapy. A subgroup analysis was further performed to evaluate the concentration distribution stratified by different types of epilepsy. Additionally, factors influencing drug exposure were analyzed using all available concentration data points from the entire cohort.

### 4.3. Efficacy Outcome Evaluation and Definitions

The seizure frequency at each time point (1, 3, 6, 12, 18, 24, 36–48, and >48 months) after starting OXC therapy was recorded, with the pre-treatment seizure frequency serving as the baseline. Under unchanged treatment regimens, therapeutic efficacy was evaluated based on the comparison between post-treatment seizure frequency and the baseline value, and defined as follows:

(1) Seizure-free: the complete absence of seizures, with a seizure-free period lasting ≥6 months at the time of follow-up; (2) response: a reduction in seizure frequency or severity by ≥50%; (3) ineffective: a reduction in seizure frequency or severity by <50%, or an increase in seizure frequency, worsening of seizure severity, or prolongation of seizure duration. The overall response rate was defined as the sum of the seizure-free rate and the response rate. This study primarily analyzed the efficacy data of OXC, either as monotherapy or add-on therapy, at 12 months, with stratification based on epilepsy type.

This study conducted an exploratory analysis of the long-term efficacy of OXC and its influencing factors, with a comprehensive evaluation using two complementary endpoints: (1) Treatment response status, defined as the clinical response at the last concentration monitoring follow-up. Patients with therapeutic efficacy categorized as “seizure-free” or “response” were classified as responders, while those with “ineffective” outcomes were defined as non-responders, thereby serving as a binary efficacy endpoint. (2) Treatment persistence, measured by “time to treatment failure,” with the endpoint event defined as any clinical decision leading to the modification of the initial OXC monotherapy regimen. This specifically includes the addition of another ASM for any clinical reason (e.g., ineffectiveness or intolerability), or the discontinuation of OXC and its replacement with a different ASM. As this endpoint assesses the sustainability of the initial monotherapy strategy, the analysis was logically restricted to the patient population who received initial OXC monotherapy rather than adjunctive therapy. This dual-endpoint design was implemented to simultaneously evaluate the long-term clinical outcomes of OXC from two dimensions: the immediate treatment response status and the long-term sustainability of the initial treatment strategy in real-world clinical practice.

We subsequently performed a correlation analysis between treatment response and exposure levels to OXC. The analysis utilized the exposure levels measured at the last concentration monitoring follow-up and their corresponding therapeutic response outcomes.

### 4.4. Safety Analysis

This study meticulously documented all adverse events identified by investigators as potentially related to OXC treatment, based on the data from HIS. Descriptive statistics were applied to analyze the reported adverse events, including the calculation of both the frequency of occurrence for each event type and the corresponding proportion of affected patients.

We systematically analyzed the time-to-onset (TTO) of adverse events potentially associated with OXC treatment. TTO was defined as the time interval between the initiation of OXC therapy and the first occurrence of a specific adverse event. This metric was used to evaluate the temporal distribution patterns of adverse events at the overall level and across different event types during OXC treatment. To further quantify the statistical distribution characteristics of TTO data and explore underlying patterns, this study employed a Weibull distribution model for fitting and interpretation [51]. The application of this model aimed to assess how the risk of adverse event occurrence changes over time (e.g., early occurrence, constant risk, or delayed onset) and to provide a theoretical basis for clinical monitoring.

The Weibull distribution is characterized by two core parameters: the scale parameter (α) and the shape parameter (β). The former reflects the spread and central tendency of the distribution; a larger α value suggests that adverse events tend to occur later in the treatment course. The latter characterizes the dynamic profile of adverse event risk over time. Based on the estimated β value and its 95% confidence interval (CI), the temporal risk patterns of adverse events were classified into three categories: (1) β > 1 with the lower limit of the 95% CI > 1 indicates a significantly increasing risk over time, exhibiting a “wear-out failure” pattern, suggestive of delayed or cumulative toxicity; (2) β < 1 with the upper limit of the 95% CI < 1 indicates a decreasing risk over time, exhibiting an “early failure” pattern, often observed in adaptive adverse reactions during the initial phase of treatment; (3) β ≈ 1 with the 95% CI including 1 indicates a constant risk over time, exhibiting a “random failure” pattern, suggesting that adverse event occurrence is independent of treatment duration, i.e., the event probability remains essentially constant during the treatment period [52,53,54].

### 4.5. Statistical Analysis

The normality of continuous variables was assessed using the Shapiro–Wilk test. Normally distributed continuous variables are presented as the mean ± standard deviation (SD), while non-normally distributed continuous variables are described as the median (interquartile range, IQR) or median (range). Categorical variables are expressed as frequency and percentage. Differences in exposure to MHD across multiple independent groups were evaluated using the Kruskal–Wallis test and Dunn’s test. Comparisons of non-normally distributed continuous variables between two groups were performed using the Mann–Whitney U test. Categorical variables were compared between groups using Pearson’s chi-square test or Fisher’s exact test. Correlations between exposure and potential influencing factors were examined using Spearman’s rank correlation analysis.

The distribution range of plasma MHD concentrations was estimated using Generalized Additive Models for Location, Scale and Shape (GAMLSS). The optimal model distribution was selected based on the minimization principle of the Akaike Information Criterion (AIC), and the plasma reference ranges of MHD were calculated for different efficacy groups and stratified by epilepsy type.

A mixed-effects model was employed to identify potential factors influencing drug exposure, accounting for bias due to repeated concentration measurements within individuals over time. The model included the following independent variables: age, body weight, the duration of epilepsy, duration of OXC therapy, sex, dosage form, the presence of gene mutation, etiology, epilepsy type, ASMs in add-on therapy, efficacy, and adverse events. The dependent variable, *C*_0_/Dose ratio, was skewed and was logarithmically transformed to meet the normality assumption. During modeling, four concentration records lacking corresponding efficacy data were excluded. The final analysis was based on 1972 blood concentration measurements from 822 pediatric epilepsy patients.

Treatment response status was evaluated at the last concentration monitoring visit. Logistic regression was used to analyze associations between variables and treatment response. The predictive performance of plasma MHD concentration for treatment response was assessed using receiver operating characteristic (ROC) curve analysis, and the optimal cutoff value was determined. Patients were subsequently divided into two groups based on this cutoff. To minimize potential confounding, propensity score matching (PSM) was performed. Matching was conducted at a 1:1 ratio using the nearest-neighbor method. A caliper of 0.2 standard deviations was applied to restrict matches, and matching was performed without replacement to ensure independence of observations. Following successful matching and verification of covariate balance, differences in treatment response were compared between the groups. Regarding the endpoint of treatment persistence, univariate and multivariate Cox proportional hazards regression models were employed in the patient population receiving initial OXC monotherapy to analyze independent predictors influencing time to treatment failure, with the aim of identifying clinical variables associated with the long-term sustainability of the OXC treatment regimen.

An exploratory analysis was conducted to examine the correlation between adverse events and drug exposure. ROC analysis was used to evaluate the predictive performance of plasma MHD concentration for the occurrence of adverse events. The cumulative incidence of adverse events was estimated using Kaplan–Meier survival curves, and the TTO data were analyzed using the Weibull shape parameter test.

All statistical analyses were performed using GraphPad Prism 10 (GraphPad Software), and R version 4.5.0. A *p*-value < 0.05 was considered statistically significant.

## 5. Conclusions

In summary, this study systematically evaluated the exposure–efficacy–safety profile of OXC in pediatric epilepsy patients within real-world clinical settings. It identified key factors influencing exposure and efficacy, revealed the temporal distribution patterns of adverse events, and provided valuable references for the rational clinical use of OXC and continuous safety monitoring. The findings indicated that the variability in plasma MHD concentrations was primarily associated with significant interindividual differences, which exerted an even greater influence than some clinically measurable factors, thereby underscoring the central importance of TDM in individualized treatment. Based on the study data, we further proposed a therapeutic reference range for MHD (3.0–20.0 µg/mL) applicable to Chinese children with epilepsy and established a tiered concentration management framework, offering actionable guidance for clinical practice. Building on the outcomes of this research, subsequent efforts may focus on developing and validating a population pharmacokinetic model for OXC, with the aim of advancing intelligent individualized dosing systems and progressively achieving the goals of precision medicine—“delivering the right dose, to the right patient, at the right time.”

## Figures and Tables

**Table 1:** Demographic and clinical characteristics of pediatric epilepsy patients included in study.

Characteristic		N (%) or Median [25th–75th Percentile]
Sex	Male/Female	494/330
Age at epilepsy onset (y)		4.77 [1.68, 7.57]
Weight (kg)		30.0 [20.0, 42.0]
Dose (mg/kg/d)		21.23 [16.98, 27.27]
Plasma MHD concentrations (µg/mL)		9.75 [7.02, 13.10]
ASMs in add-on therapy **^a^**	0	1172 (59.3)
	1	591 (29.9)
	2	164 (8.3)
	3	47 (2.4)
	4	2 (0.1)
Duration of epilepsy (months)		33.13 [16.30, 55.75]
Duration of OXC therapy (months)		24.03 [9.71, 42.43]
Abnormal EEG at baseline		416/446 (93.3)
Type of epilepsy	Focal	464 (56.3)
	Generalized	64 (7.8)
	Unknown	94 (11.4)
	Unclassified	202 (24.5)
Comorbidities	None	456 (55.3)
	Developmental delay ^b^	234 (28.4)
	ADHD	84 (10.2)
	Tic disorders	31 (3.8)
	Mental disorders ^c^	29 (3.5)
	Cardiac diseases	23 (2.8)
	Sleep disorders	9 (1.1)
	Peripheral neuropathy	5 (0.6)
	Neurovascular headache	4 (0.5)
	Obesity	18 (2.2)
	Weight loss	6 (0.7)
	Other ^d^	29 (3.5)
Etiology	Unknown	590 (71.6)
	Structural	116 (14.1)
	Genetic	67 (8.1)
	Infectious	48 (5.8)
	Metabolic	13 (1.6)
	Immune	13 (1.6)

Abbreviations: ADHD, attention-deficit and hyperactive disorder; ASM, antiseizure medicine; EEG, electroencephalography; MHD, monohydroxy derivative/10,11-dihydro-10-hydroxy carbamazepine; OXC, oxcarbazepine. Notes: N, patients; ^a^, The count of concomitant medications is based on the recorded drug regimen at each therapeutic drug monitoring follow-up visit; ^b^, Includes global developmental delay, intellectual disability, cerebral dysgenesis, motor delay, and short stature, among others; ^c^, Includes autism spectrum disorder, bipolar disorder, anxiety disorders, depressive disorders, mood disorders, and other psychiatric conditions; ^d^, Includes, but is not limited to, Langerhans cell histiocytosis, movement disorders, hemophilia, and hemiparesis/hemiplegia.

**Table 2:** Reference ranges for plasma MHD concentrations were derived using Generalized Additive Models for Location, Scale and Shape (GAMLSS) analysis across different patient populations.

	Patients	Samples	Plasma Reference Range (μg/mL)	
			P_2.5_	P_97.5_
**Seizure-free for 12 months on OXC monotherapy ^a^**				
All	254	634	3.16	27.24
Focal	131	331	3.82	23.76
Generalized	14	40	13.11	27.19
Unknown	18	57	13.92	31.15
Unclassified	91	206	4.35	24.67
**Response for 12 months on OXC monotherapy ^b^**				
All	376	942	3.75	23.33
Focal	200	493	3.82	22.94
Generalized	24	61	10.43	25.99
Unknown	30	107	4.34	32.65
Unclassified	122	281	4.35	22.15

Notes: ^a^, Patients with seizure freedom during 12 months of OXC monotherapy; ^b^, Patients with 50% or more reduction in seizure frequency/severity during 12 months of OXC monotherapy.

**Table 3:** Results of mixed-effects model analysis for plasma MHD concentrations.

Covariates		Estimate	Standard Error	T Value	*p* Value
(Intercept)		−3.316	0.102	−32.488	<0.001
Age (y)		−0.046	0.007	−6.443	<0.001 *
Weight (kg)		−0.007	0.001	−4.907	<0.001 *
Duration of epilepsy (m)		−0.001	0.001	−0.673	0.501
Duration of OXC therapy (m)		−0.002	0.001	−2.472	0.014 *
Sex	Male				
	Female	−0.018	0.027	−0.679	0.497
Dosage form	Tablets				
	Suspension	0.057	0.038	1.520	0.129
Gene mutation	Non				
	Mutation	0.037	0.071	0.522	0.602
Etiology	Genetic				
	Immune	−0.330	0.182	−1.817	0.070
	Infectious	−0.096	0.104	−0.925	0.355
	Metabolic	0.030	0.145	0.208	0.835
	Other ^a^	−0.093	0.107	−0.869	0.385
	Structural	−0.040	0.092	−0.439	0.661
	Unknown	−0.122	0.085	−1.440	0.150
Type of epilepsy	Focal				
	Generalized	−0.030	0.050	−0.594	0.553
	Unknown	−0.006	0.042	−0.143	0.887
	Unclassified	−0.092	0.033	−2.819	0.005 *
ASMs in add−on therapy	0				
	1	0.003	0.026	0.125	0.900
	≥2	−0.046	0.039	−1.172	0.241
Efficacy	Non				
	Responder	−0.036	0.031	−1.190	0.234
AE	Non				
	AE	0.051	0.029	1.769	0.077

Abbreviations: ASM, antiseizure medicine; OXC, oxcarbazepine; AE, adverse event. Notes: ^a^, Patients with dual etiology; *, *p* < 0.05.

**Table 4:** Efficacy and clinical characteristics at the last concentration monitoring follow-up.

Variables		Responders N (%)	Non-Responders N (%)	*p* Value
All patients		706 (85.9)	116 (14.1)	<0.001 *
Age (y)	Median (range)	9.25 (0.46, 17.83)	8.04 (0.75, 17.58)	0.098
Weight (kg)	Median (range)	33.0 (6.5, 110.0)	30.5 (9.5, 85.0)	0.359
Sex	Male	418 (59.2)	75 (64.7)	0.267
	Female	288 (40.8)	41 (35.3)	
Duration of epilepsy (m)	Median (range)	39.94 (0.70, 176.5)	40.29 (0.32, 138.5)	0.799
Duration of OXC therapy (m)	Median (range)	32.14 (0.10, 141.8)	26.55 (0.01, 121.8)	0.042 *
Plasma MHD concentrations (µg/mL)	Median (range)	8.95 (1.04, 29.20)	12.00 (3.01, 27.40)	<0.001 *
Dose (mg/d)	Median (range)	600.0 (60.0, 1800.0)	787.5 (120.0, 1800.0)	<0.001 *
Dosage form	Tablets	482 (68.3)	77 (66.4)	0.685
	Suspension	224 (31.7)	39 (33.6)	
Gene mutation	Mutation	77 (10.9)	14 (12.1)	0.712
	Non	629 (89.1)	102 (87.9)	
Etiology	Unknown	527 (74.6)	61 (52.6)	<0.001 *
	Structural	78 (11.0)	25 (21.6)	
	Genetic	50 (7.1)	8 (6.9)	
	Infectious	23 (3.3)	11 (9.5)	
	Metabolic	6 (0.8)	4 (3.4)	
	Other ^a^	22 (3.1)	7 (6.0)	
Type of epilepsy	Focal	389 (55.1)	73 (62.9)	0.044 *
	Generalized	57 (8.1)	7 (6.0)	
	Unknown	76 (10.8)	18 (15.5)	
	Unclassified	184 (26.1)	18 (15.5)	
ASMs in add-on therapy	0	437 (61.9)	30 (25.9)	<0.001 *
	1	201 (28.5)	45 (38.8)	
	2	54 (7.6)	31 (26.7)	
	>2	14 (2.0)	10 (8.6)	

Abbreviations: ASM, antiseizure medicine; MHD, monohydroxy derivative/10,11-dihydro-10-hydroxy carbamazepine; OXC, oxcarbazepine. Notes: N, patients; ^a^, Patients with dual etiology or immune etiology; *, *p* < 0.05.

**Table 5:** Results from multivariable logistic regression analysis of response status at last concentration monitoring follow-up.

Variables		Estimate	Standard Error	*p* Value	OR [95%CI]
(Intercept)		−4.043	0.392	<0.001	0.02 [0.01, 0.04]
Duration of OXC therapy (m)		−0.008	0.005	0.112	0.99 [0.98, 1.00]
Plasma MHD concentrations (µg/mL)		0.093	0.023	<0.001 *	1.10 [1.05, 1.15]
Dose (mg/d)		0.001	0.000	0.076	1.00 [1.00, 1.00]
Etiology	Unknown				
	Structural	0.688	0.293	0.019 *	1.99 [1.11, 3.50]
	Genetic	0.188	0.439	0.670	1.21 [0.48, 2.73]
	Infectious	1.011	0.432	0.019 *	2.75 [1.14, 6.30]
	Metabolic	1.584	0.736	0.031 *	4.87 [1.07, 20.43]
	Other ^a^	0.441	0.500	0.378	1.55 [0.55, 3.97]
ASMs in add-on therapy	0				
	1	0.965	0.262	<0.001 *	2.63 [1.58, 4.42]
	≥2	1.806	0.294	<0.001 *	6.09 [3.43, 10.91]

Abbreviations: ASM, antiseizure medicine; MHD, monohydroxy derivative/10,11-dihydro-10-hydroxy carbamazepine; OXC, oxcarbazepine. Notes: ^a^, Patients with dual etiology or immune etiology; *, *p* < 0.05.

**Table 6:** Results of univariable and multivariable Cox regression analyses in patients receiving initial OXC monotherapy.

Variables		Univariate Analysis		Variables		Multivariate Analysis	
		HR [95%CI]	*p* Value			HR [95%CI]	*p* Value
Age (y)	<9			Age (y)	<9		
	≥9	0.70 [0.53, 0.94]	0.016 *		≥9	0.88 [0.58, 1.32]	0.526
Weight (kg)	<33			Weight (kg)	<33		
	≥33	0.68 [0.51, 0.91]	0.009 *		≥33	0.75 [0.50, 1.12]	0.163
Sex	Male						
	Female	0.95 [0.71, 1.27]	0.737				
Concentrations (µg/mL)	<10			Concentrations (µg/mL)	<10		
	≥10	1.98 [1.49, 2.63]	<0.001 *		≥10	1.73 [1.29, 2.32]	<0.001 *
Dosage form	Tablets						
	Suspension	1.14 [0.82, 1.58]	0.449				
Gene mutation	Non						
	Mutation	1.39 [0.85, 2.25]	0.189				
Etiology	Unknown			Etiology	Unknown		
	Genetic	1.29 [0.63, 2.64]	0.494		Genetic	1.22 [0.59, 2.55]	0.588
	Infectious	2.56 [1.38, 4.75]	0.003 *		Infectious	2.30 [1.23, 4.31]	0.009 *
	Metabolic	3.37 [1.07, 10.64]	0.038 *		Metabolic	3.74 [1.18, 11.85]	0.025 *
	Other ^a^	2.56 [1.30, 5.05]	0.006 *		Other	1.86 [0.94, 3.71]	0.076
	Structural	1.71 [1.17, 2.49]	0.006 *		Structural	1.44 [0.98, 2.11]	0.060
Epilepsy type	Focal			Epilepsy type	Focal		
	Generalized	0.73 [0.40, 1.31]	0.290		Generalized	0.71 [0.39, 1.30]	0.266
	Unknown	1.65 [1.09, 2.48]	0.017 *		Unknown	1.58 [1.04, 2.40]	0.031 *
	Unclassified	0.44 [0.29, 0.66]	<0.001 *		Unclassified	0.49 [0.32, 0.74]	0.001 *

Abbreviations: MHD, monohydroxy derivative/10,11-dihydro-10-hydroxy carbamazepine. Notes: ^a^, Patients with dual etiology or immune etiology; *, *p* < 0.05.

**Table 7:** Number of reported adverse events.

Adverse Events	N (%)
Dizziness/Headache	88 (10.7)
Somnolence	43 (5.2)
Psychiatric symptoms ^a^	42 (5.1)
Gastrointestinal disturbance ^b^	74 (9.0)
Rash	49 (5.9)
Visual disturbances ^c^	15 (1.8)
Fatigue	12 (1.5)
Liver function abnormalities	20 (2.4)
Thyroid function abnormalities	20 (2.4)
Chest discomfort	8 (1.0)
Gait abnormality	6 (0.7)
Hyponatremia	4 (0.5)
Weight gain	2 (0.2)

Notes: N, adverse events; ^a^, includes excitement, emotional lability, irritability, agitation, aggressive behavior, depressed mood, non-suicidal self-injury, and suicidal ideation. ^b^, includes nausea, vomiting, diarrhea, abdominal pain, constipation, dyspepsia, and decreased appetite. ^c^, includes blurred vision, diplopia, ghosting images, visual haze, tunnel vision, and chromatic phosphenes.

**Table 8:** Results from Weibull distribution shape parameter hypothesis test.

Types of Adverse Events	N	α (95%CI)	β (95%CI)	Failure Pattern
Dizziness/Headache	83	659.74 (504.30, 863.10)	0.84 (0.71, 1.00)	Random failure model
Gastrointestinal disorders	74	428.29 (328.41, 558.53)	0.90 (0.76, 1.08)	Random failure model
Rash	48	126.12 (90.66, 175.44)	0.90 (0.72, 1.13)	Random failure model
Somnolence	41	528.75 (376.69, 742.20)	0.95 (0.75, 1.20)	Random failure model
Psychiatric symptoms	40	607.06 (401.01, 918.97)	0.79 (0.61, 1.01)	Random failure model
Liver function abnormalities	20	506.80 (273.72, 938.32)	0.75 (0.52, 1.08)	Random failure model
Thyroid function abnormalities	20	789.28 (495.77, 1256.55)	0.99 (0.69, 1.41)	Random failure model
Visual disorders	15	265.65 (147.60, 478.12)	0.91 (0.63, 1.33)	Random failure model
Fatigue	12	1238.24 (855.61, 1791.98)	1.58 (0.95, 2.62)	Random failure model
Chest discomfort	8	860.06 (469.60, 1575.18)	1.20 (0.67, 2.14)	Random failure model
Gait abnormality	6	1090.02 (604.28, 1966.23)	1.43 (0.76, 2.69)	Random failure model
Hyponatremia	4	511.34 (275.27, 949.88)	1.67 (0.76, 3.70)	Random failure model
Weight gain	2	586.75 (400.97, 858.62)	3.84 (1.21, 12.20)	Wear-out failure model

Notes: N, adverse events; α, scale parameter; β, shape parameter.

## Data Availability

The data presented in this study are available on request from the corresponding author due to privacy and ethical considerations related to the human participant data involved.

## Supplementary Materials

The following supporting information can be downloaded at: https://www.mdpi.com/article/10.3390/ph19030415/s1.

## References

[1] Fiest K.M., Sauro K.M., Wiebe S., Patten S.B., Kwon C.S., Dykeman J., Pringsheim T., Lorenzetti D.L., Jetté N. (2017). Prevalence and incidence of epilepsy: A systematic review and meta-analysis of international studies. Neurology.

[2] Ioannou P., Foster D.L., Sander J.W., Dupont S., Gil-Nagel A., Drogon O’Flaherty E., Alvarez-Baron E., Medjedovic J. (2022). The burden of epilepsy and unmet need in people with focal seizures. Brain Behav..

[3] Tandon N., Radosavljevic M., Vucevic D., Radenkovic M., Jancic J., Samardzic J. (2024). Anti-seizure Medications: Challenges and Opportunities. CNS Neurol. Disord. Drug Targets.

[4] Koch M.W., Polman S.K. (2009). Oxcarbazepine versus carbamazepine monotherapy for partial onset seizures. Cochrane Database Syst. Rev..

[5] Nevitt S.J., Tudur Smith C., Marson A.G. (2018). Oxcarbazepine versus phenytoin monotherapy for epilepsy: An individual participant data review. Cochrane Database Syst. Rev..

[6] Liu Y.J., Guo H.L., Zhang G., Xu J., Wu C.F., Chen F. (2025). Clinical rational application of oxcarbazepine in epilepsy—Recommendation from guidelines and consensus. Chin. J. Hosp. Pharm..

[7] May T.W., Korn-Merker E., Rambeck B. (2003). Clinical pharmacokinetics of oxcarbazepine. Clin. Pharmacokinet..

[8] Flesch G. (2004). Overview of the clinical pharmacokinetics of oxcarbazepine. Clin. Drug Investig..

[9] Patsalos P.N., Spencer E.P., Berry D.J. (2018). Therapeutic Drug Monitoring of Antiepileptic Drugs in Epilepsy: A 2018 Update. Ther. Drug Monit..

[10] Lim S.N., Wu T., Chang C.W., Johnny Tseng W.E., Cheng M.Y., Hsieh H.Y., Lee C.H., Lin W.R., Liu C.J., Chen P.R. (2024). Clinical impact of therapeutic drug monitoring for newer anti-seizure medications in patients with epilepsy: A real-world observation study. Biomed. J..

[11] Sattler A., Schaefer M., May T.W. (2015). Relationship between mono-hydroxy-carbazepine serum concentrations and adverse effects in patients on oxcarbazepine monotherapy. Seizure.

[12] Ji Z., Li T., Zhao X., Ma W., Li Y., Huang J. (2022). Development and Validation of a Highly Sensitive and Rapid LC-MS(3) Strategy to Determine Oxcarbazepine and Its Active Metabolite in the Serum of Patients with Epilepsy and Its Application in Therapeutic Drug Monitoring. Molecules.

[13] Friis M.L., Kristensen O., Boas J., Dalby M., Deth S.H., Gram L., Mikkelsen M., Pedersen B., Sabers A., Worm-Petersen J. (1993). Therapeutic experiences with 947 epileptic out-patients in oxcarbazepine treatment. Acta Neurol. Scand..

[14] Patsalos P.N., Berry D.J., Bourgeois B.F., Cloyd J.C., Glauser T.A., Johannessen S.I., Leppik I.E., Tomson T., Perucca E. (2008). Antiepileptic drugs—Best practice guidelines for therapeutic drug monitoring: A position paper by the subcommission on therapeutic drug monitoring, ILAE Commission on Therapeutic Strategies. Epilepsia.

[15] Chen C.Y., Zhou Y., Cui Y.M., Yang T., Zhao X., Wu Y. (2019). Population pharmacokinetics and dose simulation of oxcarbazepine in Chinese paediatric patients with epilepsy. J. Clin. Pharm. Ther..

[16] Wu W., Yang W.S., Xu X.Y., Ge X.L., Lu J., Wang G.F., Wang Y., Li Z.P. (2024). Population pharmacokinetics of oxcarbazepine active metabolite in Chinese paediatric patients with epilepsy: Model-based dose optimization. Basic Clin. Pharmacol. Toxicol..

[17] Yao N., Huang S., Huang A., Song H. (2022). Analysis of influencing factors on monohydroxylated derivative of oxcarbazepine plasma concentration in children with epilepsy. Eur. J. Clin. Pharmacol..

[18] Qin Y., Zhang N., Yang Y., Teng Y., Xia Z., Mao Z., Zhang P., Niu W. (2025). Optimizing oxcarbazepine therapy for epilepsy in patients aged 0–16 years: A comprehensive study of individualized treatment factors. Eur. J. Pharmacol..

[19] Striano S., Striano P., Di Nocera P., Italiano D., Fasiello C., Ruosi P., Bilo L., Pisani F. (2006). Relationship between serum mono-hydroxy-carbazepine concentrations and adverse effects in patients with epilepsy on high-dose oxcarbazepine therapy. Epilepsy Res..

[20] Rodrigues C., Chiron C., Rey E., Dulac O., Comets E., Pons G., Jullien V. (2017). Population pharmacokinetics of oxcarbazepine and its monohydroxy derivative in epileptic children. Br. J. Clin. Pharmacol..

[21] Reimers A., Berg J.A., Burns M.L., Brodtkorb E., Johannessen S.I., Johannessen Landmark C. (2018). Reference ranges for antiepileptic drugs revisited: A practical approach to establish national guidelines. Drug Des. Dev. Ther..

[22] Beydoun A., DuPont S., Zhou D., Matta M., Nagire V., Lagae L. (2020). Current role of carbamazepine and oxcarbazepine in the management of epilepsy. Seizure.

[23] Chen Y.T., Wang C.Y., Yin Y.W., Li Z.R., Lin W.W., Zhu M., Jiao Z. (2021). Population pharmacokinetics of oxcarbazepine: A systematic review. Expert. Rev. Clin. Pharmacol..

[24] Peng Q., Ma M., Gu X., Hu Y., Zhou B. (2021). Evaluation of Factors Impacting the Efficacy of Single or Combination Therapies of Valproic Acid, Carbamazepine, and Oxcarbazepine: A Longitudinal Observation Study. Front. Pharmacol..

[25] Italiano D., Perucca E. (2013). Clinical pharmacokinetics of new-generation antiepileptic drugs at the extremes of age: An update. Clin. Pharmacokinet..

[26] Armijo J.A., Vega-Gil N., Shushtarian M., Adín J., Herranz J.L. (2005). 10-Hydroxycarbazepine serum concentration-to-oxcarbazepine dose ratio: Influence of age and concomitant antiepileptic drugs. Ther. Drug Monit..

[27] Malátková P., Havlíková L., Wsól V. (2014). The role of carbonyl reducing enzymes in oxcarbazepine in vitro metabolism in man. Chem. Biol. Interact..

[28] Yuan Y., Zhang S., Yuan Y., Yan X., Zhang L., Ran Y.W. (2023). Pharmacogenomics of oxcarbazepine in the treatment of epilepsy. Pharmacogenomics.

[29] Högler W., Wudy S.A., Luef G., Hartmann M.F., Rauchenzauner M. (2010). Oxcarbazepine accelerates cortisol elimination via cytochrome P450 3A4 induction. Arch. Dis. Child..

[30] Sugiyama I., Murayama N., Kuroki A., Kota J., Iwano S., Yamazaki H., Hirota T. (2016). Evaluation of cytochrome P450 inductions by anti-epileptic drug oxcarbazepine, 10-hydroxyoxcarbazepine, and carbamazepine using human hepatocytes and HepaRG cells. Xenobiotica.

[31] Shen X., Zhu J., He Y., Wang X., Li W., Fan X. (2025). ABCC2 as a novel covariate influencing oxcarbazepine pharmacokinetics in pediatric epilepsy: Insights from population modeling. Drug Metab. Dispos..

[32] Wang Y., Chen Y.B., Zhang Y.Q., Luo R., Wang H., Lv J.L., Wang D., Zhu S.Q., Lin Z.D., Qin J. (2017). Oxcarbazepine oral suspension in pediatric patients with partial seizures and/or generalized tonic-clonic seizures: A multi-center, single arm, observational study in China. World J. Pediatr..

[33] Kanner A.M., Ashman E., Gloss D., Harden C., Bourgeois B., Bautista J.F., Abou-Khalil B., Burakgazi-Dalkilic E., Llanas Park E., Stern J. (2018). Practice guideline update summary: Efficacy and tolerability of the new antiepileptic drugs I: Treatment of new-onset epilepsy: Report of the Guideline Development, Dissemination, and Implementation Subcommittee of the American Academy of Neurology and the American Epilepsy Society. Neurology.

[34] Zhu H., Deng X., Feng L., Lian Y., Han X., Guo Z., Gou Y., Du Y., Xie L., Yao D. (2022). Efficacy comparison of oxcarbazepine and levetiracetam monotherapy among patients with newly diagnosed focal epilepsy in China: A multicenter, open-label, randomized study. CNS Neurosci. Ther..

[35] Ma Y., Deng J., Fu Z., Chen C., Wang X., Wang X., Weng J., Shen Y., Wang X., Fang F. (2023). Efficacy and tolerability of oxcarbazepine in the treatment of focal epilepsy in neonates and infants under 3 months of age: A single-center retrospective analysis. Epilepsy Res..

[36] Kanner A.M., Ashman E., Gloss D., Harden C., Bourgeois B., Bautista J.F., Abou-Khalil B., Burakgazi-Dalkilic E., Llanas Park E., Stern J. (2018). Practice guideline update summary: Efficacy and tolerability of the new antiepileptic drugs II: Treatment-resistant epilepsy: Report of the Guideline Development, Dissemination, and Implementation Subcommittee of the American Academy of Neurology and the American Epilepsy Society. Neurology.

[37] Deng N.J., Li X.Y., Zhang Z.X., Xian-Yu C.Y., Tao Y.T., Ma Y.T., Li H.J., Gao T.Y., Liu X., Luo J. (2025). Effectiveness and safety of single anti-seizure medication as adjunctive therapy for drug-resistant focal epilepsy based on network meta-analysis. Front. Pharmacol..

[38] Vezzani A., Fujinami R.S., White H.S., Preux P.M., Blümcke I., Sander J.W., Löscher W. (2016). Infections, inflammation and epilepsy. Acta Neuropathol..

[39] Hinojosa-Figueroa M.S., Cruz-Caraguay M., Torres Pasquel A., Puga Rosero V., Eguiguren Chavez C.B., Rodas J.A., Leon-Rojas J.E. (2025). Etiologies of Multidrug-Resistant Epilepsy in Latin America: A Comprehensive Review of Structural, Genetic, Metabolic, Inflammatory, and Infectious Origins: A Systematic Review. Biomolecules.

[40] Villanueva V., Majid O., Nabangchang C., Yang H., Laurenza A., Ferry J., Hussein Z. (2016). Pharmacokinetics, exposure-cognition, and exposure-efficacy relationships of perampanel in adolescents with inadequately controlled partial-onset seizures. Epilepsy Res..

[41] Chen Z., Brodie M.J., Liew D., Kwan P. (2018). Treatment Outcomes in Patients with Newly Diagnosed Epilepsy Treated with Established and New Antiepileptic Drugs: A 30-Year Longitudinal Cohort Study. JAMA Neurol..

[42] Egesa I.J., Newton C., Kariuki S.M. (2022). Evaluation of the International League Against Epilepsy 1981, 1989, and 2017 classifications of seizure semiology and etiology in a population-based cohort of children and adults with epilepsy. Epilepsia Open.

[43] Qin J., Wang Y., Huang X.F., Zhang Y.Q., Fang F., Chen Y.B., Lin Z.D., Deng Y.C., Yin F., Jiang L. (2018). Oxcarbazepine oral suspension in young pediatric patients with partial seizures and/or generalized tonic-clonic seizures in routine clinical practice in China: A prospective observational study. World J. Pediatr..

[44] Berghuis B., Hulst J., Sonsma A., McCormack M., de Haan G.J., Sander J.W., Lindhout D., Koeleman B.P.C. (2021). Symptomatology of carbamazepine- and oxcarbazepine-induced hyponatremia in people with epilepsy. Epilepsia.

[45] Čiauškaitė J., Gelžinienė G., Jurkevičienė G. (2022). Oxcarbazepine and Hyponatremia. Medicina.

[46] Phillips E.J., Sukasem C., Whirl-Carrillo M., Müller D.J., Dunnenberger H.M., Chantratita W., Goldspiel B., Chen Y.T., Carleton B.C., George A.L. (2018). Clinical Pharmacogenetics Implementation Consortium Guideline for HLA Genotype and Use of Carbamazepine and Oxcarbazepine: 2017 Update. Clin. Pharmacol. Ther..

[47] Lee J.E., Min K.R., Kim S.H., Kim A.H., Kim S.T. (2020). Analysis of Adverse Drug Reactions with Carbamazepine and Oxcarbazepine at a Tertiary Care Hospital. Yonsei Med. J..

[48] Li J., Zhong R., Zhang F., Guo Y. (2025). Psychiatric disorders associated with newer antiseizure medications: A real-world disproportionality analysis of FDA adverse event reporting system. Epilepsy Behav..

[49] Beniczky S., Trinka E., Wirrell E., Abdulla F., Al Baradie R., Alonso Vanegas M., Auvin S., Singh M.B., Blumenfeld H., Bogacz Fressola A. (2025). Updated classification of epileptic seizures: Position paper of the International League Against Epilepsy. Epilepsia.

[50] Zhang Y.Y., Xia Y., Guo H.L., Hu Y.H., Wen X.Y., Chen J., Lu X.P., Wang S.S., Qiu J.C., Chen F. (2022). An LC-ESI-MS/MS assay for the therapeutic drug monitoring of 15 antiseizure medications in plasma of children with epilepsy. Biomed. Chromatogr..

[51] Cornelius V.R., Sauzet O., Evans S.J. (2012). A signal detection method to detect adverse drug reactions using a parametric time-to-event model in simulated cohort data. Drug Saf..

[52] Sauzet O., Carvajal A., Escudero A., Molokhia M., Cornelius V.R. (2013). Illustration of the weibull shape parameter signal detection tool using electronic healthcare record data. Drug Saf..

[53] Kinoshita S., Hosomi K., Yokoyama S., Takada M. (2020). Time-to-onset analysis of amiodarone-associated thyroid dysfunction. J. Clin. Pharm. Ther..

[54] He W., Tong L., Yuan Y., Yang X., Yang W., Pan X. (2025). Adverse drug reactions to atezolizumab in combination with bevacizumab in hepatocellular carcinoma patients: An analysis of the food and drug administration adverse event reporting system database. Front. Pharmacol..

