# Supplementary material for: Stratified Therapeutic Drug Monitoring Could Potentially Improve the Efficacy and Safety of Oxcarbazepine in Children with Epilepsy: Novel Insights from a Single-Center, Large-Sample, Retrospective Real-World Study
Source: Pharmaceuticals (Basel). 2026 Mar 3;19(3):415. doi: 10.3390/ph19030415 (PMC13029625; doi:10.3390/ph19030415)

Stratified therapeutic drug monitoring could improve efficacy and safety of oxcarbazepine in children with epilepsy: Novel insights from a single-center, large-sample, retrospective real-world study

Yi-Jing Liu[1,2, †], Xi-Li Sun[1], Yue Li[1], Xiao-Peng Lu[3], Chun-Feng Wu[3, *], Hu Guo[3, *], Feng Chen[1, *]

[1]Pharmaceutical Sciences Research Center, Department of Pharmacy, Children's Hospital of Nanjing Medical University, Nanjing, China

[2]School of Basic Medicine and Clinical Pharmacy, China Pharmaceutical University, Nanjing, China

[3]Department of Neurology, Children's Hospital of Nanjing Medical University, Nanjing, China

[†]Visiting graduate student from China Pharmaceutical University

*Corresponding authors at Children's Hospital of Nanjing Medical University, 72 Guangzhou Road, Nanjing 210008, China.

E-mail addresses: cy.chen508@gmail.com (F. Chen), drhguo@163.com (H. Guo), and chunfeng@2008.sina.com (C.F. Wu).

----Supplemental Material---

Supplementary Table S1 Comparison of demographic and clinical characteristics before and after propensity score matching (PSM).

Variables		Before propensity score matching			After propensity score matching		
		**<10 µg/mL (N=467)**	**≥10 µg/mL (N=355)**	**P value**	**<10 µg/mL** (N=326)	**≥10 µg/mL** (N=326)	**P value**
Age (y)	Median [Range]	8.92 [0.500,	9.08 [0.460, 17.8]	0.292	9.38 [0.500, 17.8]	9.29 [0.460, 17.8]	0.753
Weight (kg)	Median [Range]	31.0 [6.50, 90.0]	34.0 [7.00, 110]	0.065	35.0 [7.00, 90.0]	35.0 [7.00, 110]	0.747
Sex	Male	272 (58.2%)	221 (62.3%)	0.276	198 (60.7%)	201 (61.7%)	0.872
	Female	195 (41.8%)	134 (37.7%)		128 (39.3%)	125 (38.3%)	
Duration of epilepsy (m)	Median [Range]	40.3 [0.700, 176]	39.8 [0.320, 172]	0.321	43.1 [1.63, 151]	39.0 [1.13, 172]	0.838
Duration of OXC therapy (m)	Median [Range]	31.5 [0.570, 142]	30.2 [0.0100, 104]	0.856	33.0 [0.670, 140]	30.7 [0.100, 104]	0.953
Gene mutation	Mutation	50 (10.7%)	41 (11.5%)	0.788	37 (11.3%)	32 (9.8%)	0.611
	Non	417 (89.3%)	314 (88.5%)		289 (88.7%)	294 (90.2%)	
Dosage form	Tablets	307 (65.7%)	252 (71.0%)	0.128	232 (71.2%)	235 (72.1%)	0.862
	Suspension	160 (34.3%)	103 (29.0%)		94 (28.8%)	91 (27.9%)	
Etiology	Unknown	348 (74.5%)	240 (67.6%)	0.019*	231 (70.9%)	227 (69.6%)	0.752
	Structural	48 (10.3%)	55 (15.5%)		43 (13.2%)	47 (14.4%)	
	Genetic	37 (7.9%)	21 (5.9%)		24 (7.4%)	19 (5.8%)	
	Other [a]	34 (7.3%)	39 (11.0%)		28 (8.6%)	33 (10.1%)	
Type of epilepsy	Focal	246 (52.7%)	216 (60.8%)	0.004*	197 (60.4%)	195 (59.8%)	0.965
	Generalized	38 (8.1%)	26 (7.3%)		24 (7.4%)	26 (8.0%)	
	Unknown	47 (10.1%)	47 (13.2%)		37 (11.3%)	40 (12.3%)	
	Unclassified	136 (29.1%)	66 (18.6%)		68 (20.9%)	65 (19.9%)	
ASMs in add-on therapy	0	308 (66.0%)	159 (44.8%)	< 0.001*	170 (52.1%)	159 (48.8%)	0.469
	1	111 (23.8%)	135 (38.0%)		109 (33.4%)	126 (38.7%)	
	2	41 (8.8%)	44 (12.4%)		40 (12.3%)	37 (11.3%)	
	> 2	7 (1.5%)	17 (4.8%)		7 (2.1%)	4 (1.2%)	

Abbreviations: ASM, antiseizure medicine; OXC, oxcarbazepine. **Notes:** [a], Patients with infectious etiology, metabolic etiology, immune etiology, or dual etiology; *, P < 0.05.

Figure legends

Supplementary Figure S1 Residual diagnostic plots of the mixed-effects model. **(A)** Linearity diagnostic plot (residuals vs. fitted values): Examines the linear relationship and homoscedasticity of the model. Ideally, residuals should be randomly distributed around the red dashed line (y=0), with no apparent trends or heteroscedasticity; **(B)** Residual Q-Q plot: Assesses the normality of residuals. If the data points (blue) closely follow the red reference line, the residuals are normally distributed; deviations suggest potential skewness or outliers; **(C)** Residual density plot: Compares the kernel density curve (blue) with the theoretical normal distribution (red dashed line) to further evaluate the distributional characteristics of the residuals.

Supplementary Figure S2 Kaplan-Meier survival curve for time to treatment failure in 575 patients receiving initial OXC monotherapy (median survival time: 73.2 months).

Supplementary Figure S3 Forest plot for time to treatment failure in 575 patients receiving initial OXC monotherapy. Hazard ratios (HRs) with 95% confidence intervals (CIs) are shown. The vertical dashed line indicates the null effect (HR = 1). *, $P < 0.05$; **, $P < 0.01$; ***, $P < 0.001$.

Supplementary Figure S4 Analysis of adverse events and plasma MHD concentration C_0 (μg/mL). **(A)** Comparison of MHD C_0 between patients with adverse events (AEs) and non-AEs among all patients (824 patients, 1976 concentrations); **(B)** Comparison of MHD C_0 between patients with adverse events (AEs) and non-AEs among patients receiving OXC monotherapy throughout the course (387 patients, 915 concentrations); **(C)** The ROC curve demonstrates the predictive performance of plasma MHD concentration for adverse events (824 patients, 1976 concentrations). The optimal cutoff value was 8.68 μg/mL (sensitivity = 66.4%, specificity = 42.9%). The area under the curve (AUC) was 0.55 (95% CI: 0.52–0.57, $P < 0.001$). The black dashed line indicates random prediction (AUC = 0.5); **(D)** The ROC curve demonstrates the predictive performance of plasma MHD concentration for adverse events (387 patients, 915 concentrations). The optimal cutoff value was 8.68 μg/mL (sensitivity = 60.8%, specificity = 54.1%). The area under the curve (AUC) was 0.57 (95% CI: 0.53–0.61, $P = 0.001$). The black dashed line indicates random prediction (AUC = 0.5).

Supplementary Figure S1

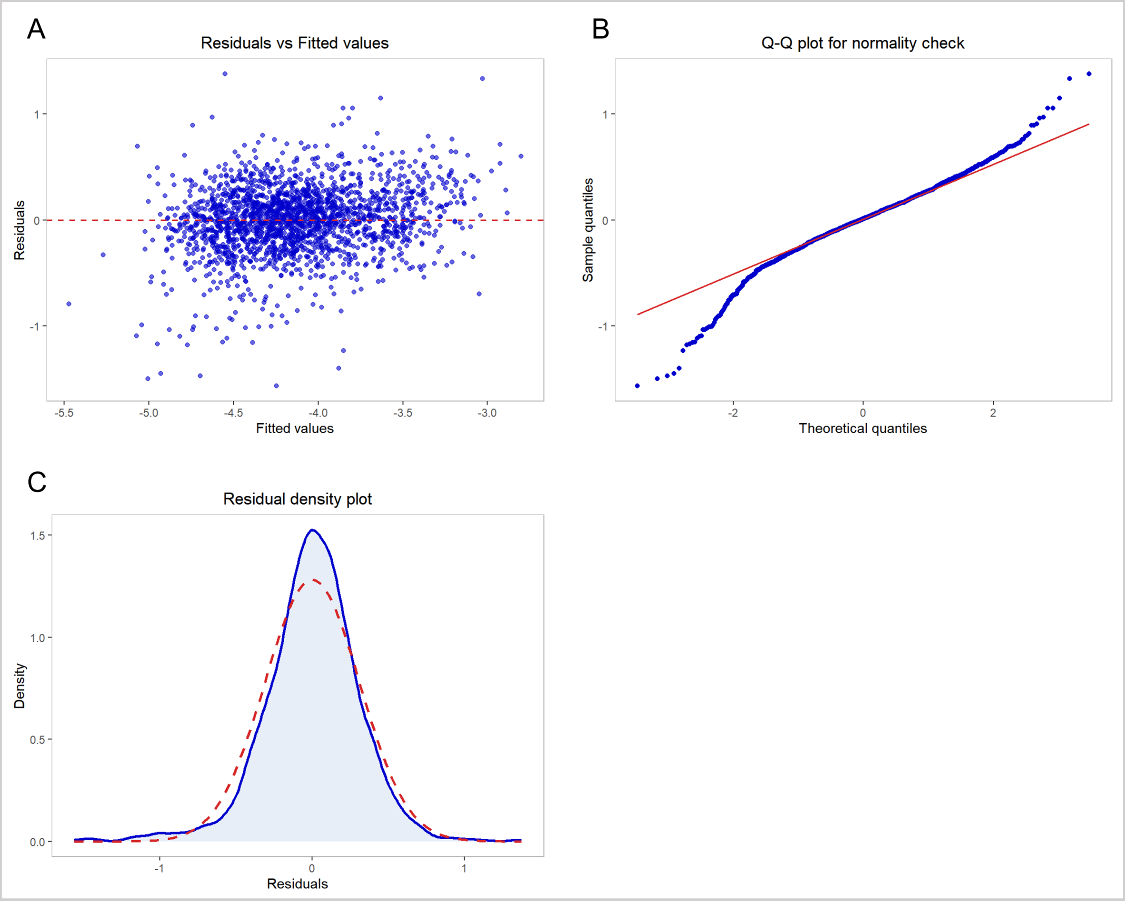

Supplementary Figure S2

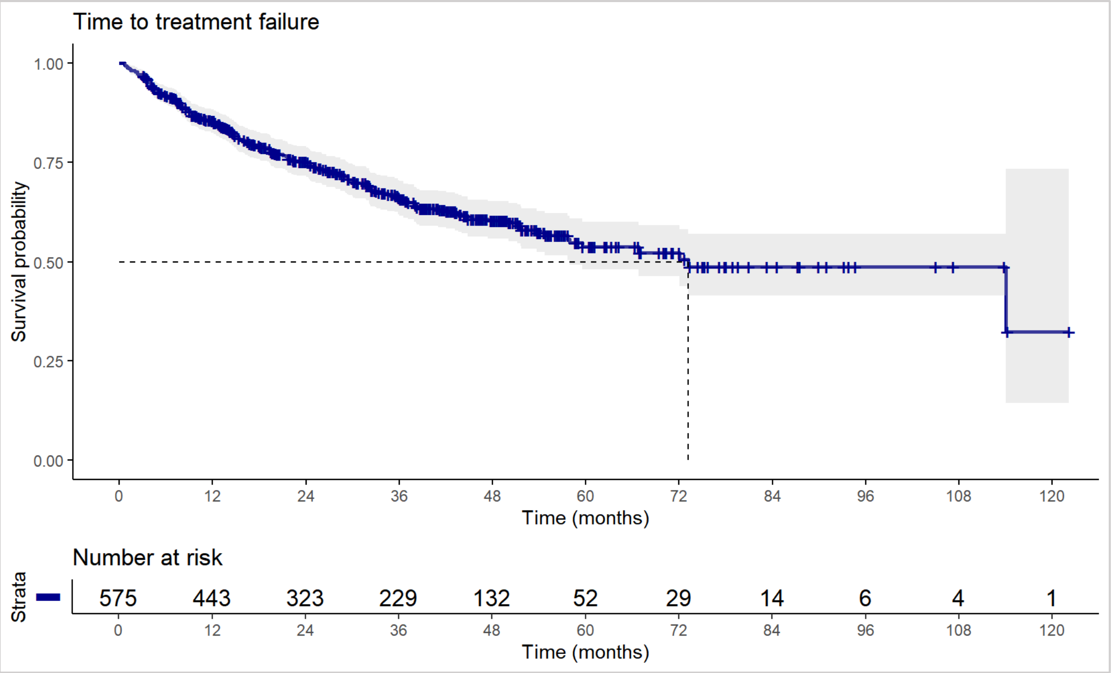

Supplementary Figure S3

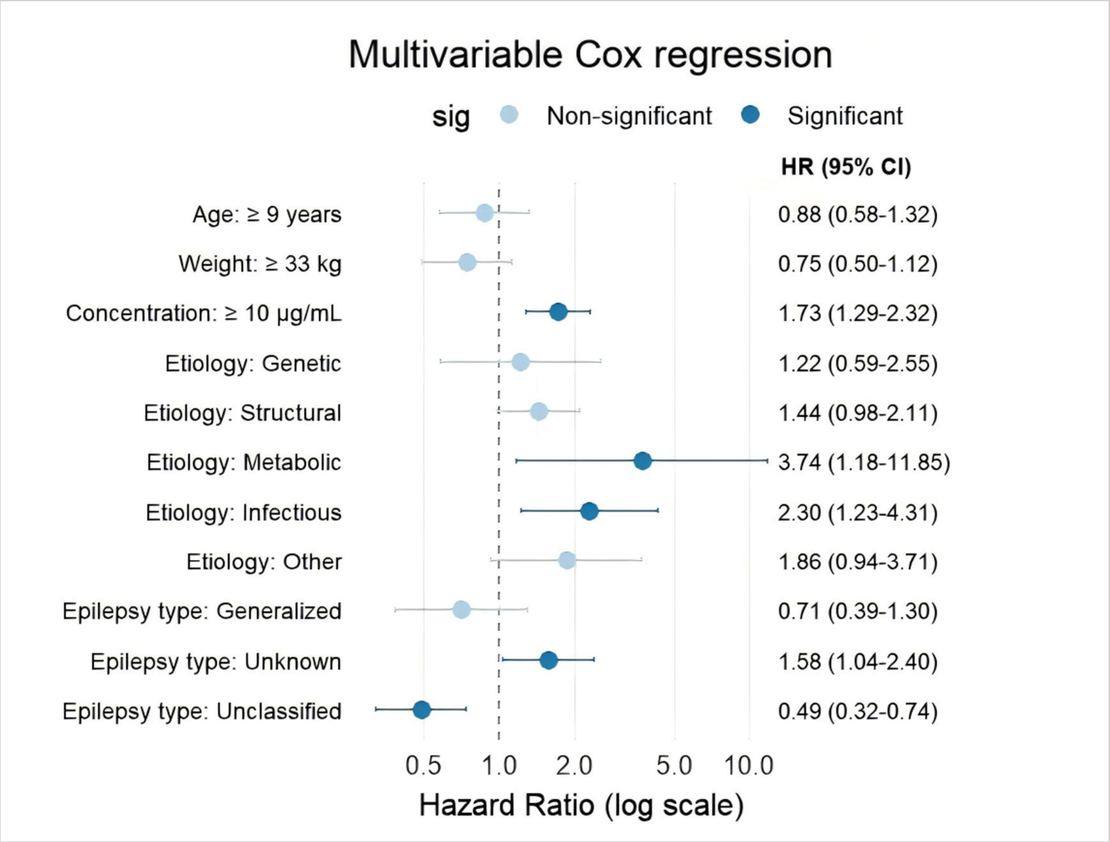

Supplementary Figure S4

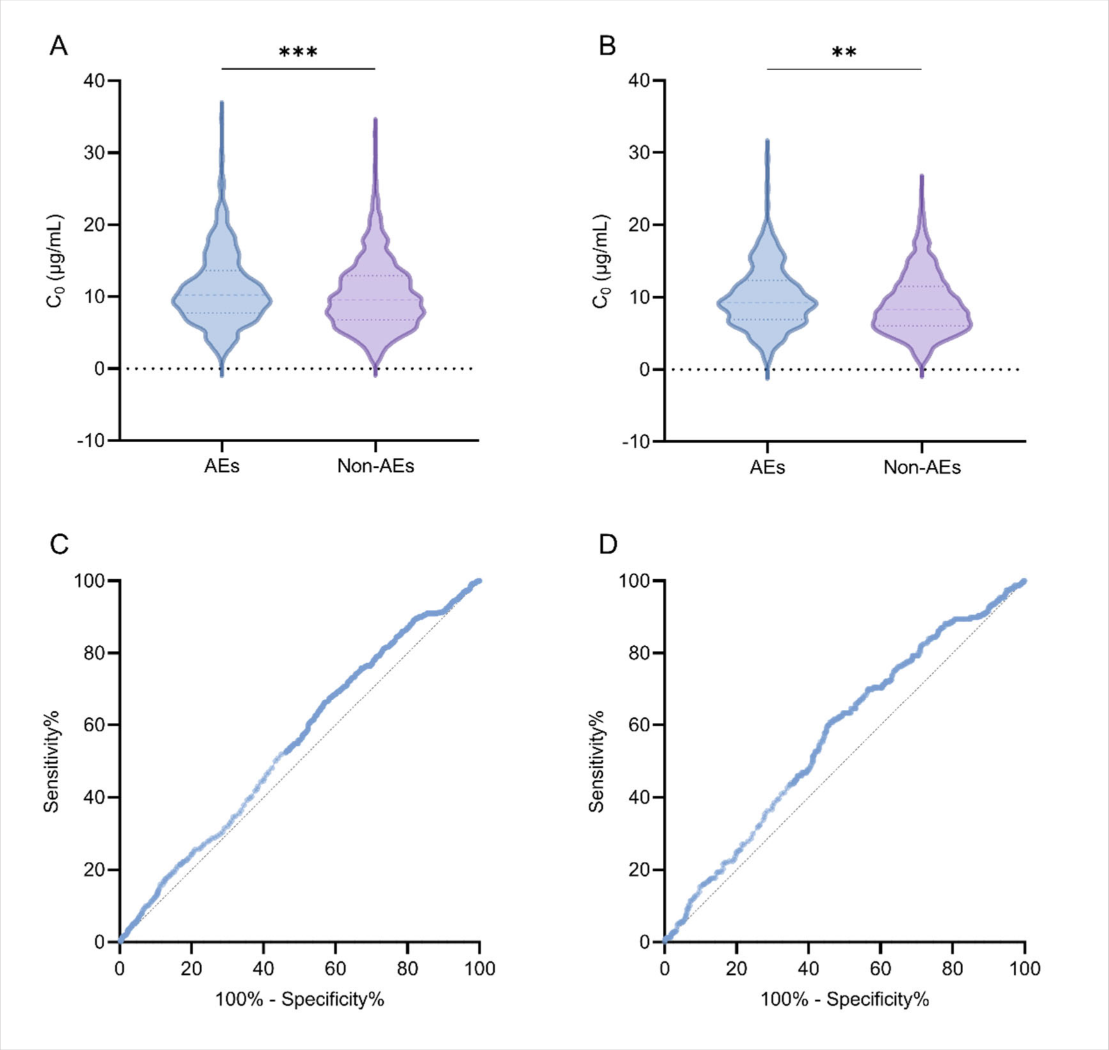

Supplement: Supplementary file 1 [file pharmaceuticals-19-00415-s001.zip › pharmaceuticals-4107207-supplementary.pdf]
